# Supplementary material for: Mortality in patients with adrenal insufficiency: a protocol for a systematic review and meta-analysis
Source: BMJ Open. 2024 Jan 29;14(1):e076582. doi: 10.1136/bmjopen-2023-076582 (PMC10826553; doi:10.1136/bmjopen-2023-076582)
Supplement: Author's manuscript [file bmjopen-2023-076582.draft_revisions.pdf]

BMJ Open is committed to open peer review. As part of this commitment we make the peer review history of every article we publish publicly available.

When an article is published we post the peer reviewers' comments and the authors' responses online. We also post the versions of the paper that were used during peer review. These are the versions that the peer review comments apply to.

The versions of the paper that follow are the versions that were submitted during the peer review process. They are not the versions of record or the final published versions. They should not be cited or distributed as the published version of this manuscript.

BMJ Open is an open access journal and the full, final, typeset and author-corrected version of record of the manuscript is available on our site with no access controls, subscription charges or pay-per-view fees (http://bmjopen.bmj.com).

If you have any questions on BMJ Open's open peer review process please email info.bmjopen@bmj.com

Mortality in patients with adrenal insufficiency: a protocol for a systematic review and meta-analysis

Journal:	*BMJ Open*
Manuscript ID	bmjopen-2023-076582
Article Type:	Protocol
Date Submitted by the Author:	11-Jun-2023

Complete List of Authors:	Allosso, Francesca; University of Campania Luigi Vanvitelli, Department of Advanced Medical and Surgical Sciences<br>Dalakas, Konstantinos; University of Gothenburg, Department of Internal Medicine and Clinical Nutrition; Sahlgrenska University Hospital, Department of Endocrinology<br>Bergthorsdottir, Ragnhildur; University of Gothenburg, Department of Internal Medicine and Clinical Nutrition; Sahlgrenska University Hospital, Department of Endocrinology<br>Chantzichristos, Dimitrios; University of Gothenburg, Department of Internal Medicine and Clinical Nutrition; Sahlgrenska University Hospital, Department of Endocrinology<br>Hessman, Eva; University of Gothenburg, Biomedical Library, Gothenburg University Library<br>Nwaru, Bright; University of Gothenburg Institute of Medicine, Krefting Research Centre; University of Gothenburg Wallenberg Centre for Molecular and Translational Medicine<br>Bobbio, Emanuele; Sahlgrenska University Hospital, Department of Cardiology; University of Gothenburg,<br>Pasquali, Daniela; University of Campania Luigi Vanvitelli, Department of Advanced Medical and Surgical Sciences<br>Johannsson, Gudmundur; University of Gothenburg, Department of Internal Medicine and Clinical Nutrition; Sahlgrenska University Hospital, Department of Endocrinology<br>Esposito, Daniela; University of Gothenburg, Department of Internal Medicine and Clinical Nutrition, Institute of Medicine, Sahlgrenska Academy; Sahlgrenska University Hospital, Department of Endocrinology

Keywords:	Pituitary disorders < DIABETES & ENDOCRINOLOGY, Adrenal disorders < DIABETES & ENDOCRINOLOGY, General endocrinology < DIABETES & ENDOCRINOLOGY, CARDIOLOGY

SCHOLARONE™ Manuscripts

Mortality in patients with adrenal insufficiency: a protocol for a systematic review and meta-analysis

Francesca Allosso*[1], Konstantinos Dalakas*[2,3], Ragnhildur Bergthorsdottir[2,3], Dimitrios Chantzichristos[2,3], Eva Hessman[4], Bright I. Nwaru[5,6], Emanuele Bobbio[7,8], Daniela Pasquali[1], Gudmundur Johannsson[2,3], Daniela Esposito[2,3]

*These authors contributed equally

Affiliation:

[1]Department of Advanced Medical and Surgical Sciences, University of Campania "Luigi Vanvitelli", Naples, Italy; [2] Department of Internal Medicine and Clinical Nutrition, Institute of Medicine, Sahlgrenska Academy, University of Gothenburg, Gothenburg, Sweden; [3]Department of Endocrinology, Sahlgrenska University Hospital, Gothenburg, Sweden; [4]Biomedical Library, Gothenburg University Library, University of Gothenburg, Gothenburg, Sweden; [5] Krefting Research Centre, Institute of Medicine, Sahlgrenska Academy, University of Gothenburg, Gothenburg, Sweden; [6] Wallenberg Centre for Molecular and Translational Medicine, Institute of Medicine, University of Gothenburg, Gothenburg, Sweden; [7]Department of Cardiology, Sahlgrenska University Hospital, Gothenburg, Sweden; [8]Institute of Medicine at Sahlgrenska Academy, University of Gothenburg, Gothenburg, Sweden

Key Words: adrenal insufficiency, hypopituitarism, mortality, glucocorticoid replacement

Abstract: words 236

Manuscript: 2215 words

References: 24

Running title: Mortality in adrenal insufficiency

For peer review only - http://bmjopen.bmj.com/site/about/guidelines.xhtml

Corresponding author and person to whom reprint requests should be addressed:
Daniela Esposito, MD, PhD, Department of Endocrinology, Sahlgrenska University Hospital Institute of Medicine, Sahlgrenska Academy, University of Gothenburg, Gothenburg, Sweden. Email: daniela.esposito@gu.se

For peer review only

For peer review only - http://bmjopen.bmj.com/site/about/guidelines.xhtml

Abstract

Introduction

Adrenal insufficiency (AI) is a rare disorder characterized by an impaired secretion of glucocorticoids from the adrenal glands. Treatment strategies for AI have developed over time with reduced glucocorticoid replacement doses and improved circadian exposure regimens, but whether this has resulted in better survival is unknown.

The main purpose of this systematic review is to gather and synthesize available evidence on mortality in patients with AI. Secondary aim is to study causes of death, with focus on cardiovascular and infectious diseases, and time trends in mortality in AI patients.

Methods and analysis

Studies published from the inception of respective databases (Medline, Embase, Cochrane and Web of Science) until the end of May 2023 will be systematically synthetized. Observational studies with a reference population will be included, and their quality will be assessed using the Newcastle-Ottawa scale. Data collected will be narratively integrated and a meta-analysis will be performed to pool data from studies considered homogenous. The systematic review will be reported following the PRISMA guidelines. This will be the first systematic review assessing mortality, causes of death and time trends in mortality in AI patients. The findings of this systematic review will be of value for both patients and healthcare providers.

Ethics and dissemination

This systematic review does not require ethical approval or informed consent because it will be based on previously published data only and does not implicate any direct contact with individual patients.

PROSPERO registration number: CRD42023416253

For peer review only - http://bmjopen.bmj.com/site/about/guidelines.xhtml

Strengths and limitations of this study

- This systematic review will for the first time provide a comprehensive synthesis of overall mortality and time trends in mortality in patients with adrenal insufficiency.
- The protocol of this systematic review follows the Preferred Reporting Items for Systematic Review and Meta-Analysis Protocols guidelines.
- A detailed description of the review processes is presented in this protocol increasing the reproducibility of the systematic review.
- All available studies on mortality in adrenal insufficiency will be gathered from publicly available literature databases, without language or geographical limitations.
- A potential limitation can be high heterogeneity in the population, as both primary and secondary adrenal insufficiency are going to be included. However, separate sub-group analyses will be performed.

For peer review only

Introduction

Adrenal insufficiency (AI) is a rare disorder characterized by an impaired secretion of glucocorticoids from the adrenal cortex (1). Primary AI (PAI), with an estimated prevalence of 93-144/million in Europe, is caused by disorders of the adrenal glands and is usually associated with mineralocorticoid and androgen deficiency (1). It can be acquired or inherited as in congenital adrenal hyperplasia (CAH), usually due to steroid 21-hydroxylase deficiency (2). In western countries acquired PAI is most commonly due to an autoimmune-mediated adrenalitis, which can be isolated or combined with other autoimmune diseases (e.g. autoimmune polyglandular syndrome type 1 or 2)(2, 3). Other causes of PAI are tuberculosis and infectious diseases (3). Secondary AI (SAI), with an estimated prevalence of 150-280/million, is caused by adrenocorticotropic hormone (ACTH) deficiency secondary to a pituitary disease and is often associated with other pituitary hormone deficiencies (1, 2).

Treatment of AI consists of replacement therapy with glucocorticoids (GC) (1). In PAI, mineralocorticoid replacement therapy is also required while in SAI additional replacement therapy with thyroid hormone, sex steroids and growth hormone may be needed due to failure of other pituitary hormone axes (2). Before the introduction of GC therapy, most patients with PAI died within 2 years of diagnosis (4). Although long-term outcomes have markedly improved, mortality remains increased, with cardiovascular disease being the leading cause of death (5-7).

Standard replacement treatments in AI include short-acting glucocorticoids such as hydrocortisone and cortisone acetate, usually given 2-3 times daily (8). In some countries other GCs such as prednisolone are used (2). However, conventional GC replacement fails to restore a circadian cortisol profile in patients with AI.

There have been attempts to improve the dosing regimen of GC replacement therapy and the management of intercurrent illnesses (9). The introduction of new therapies, as once-daily modified release hydrocortisone that better mimics the endogen circadian production of cortisol, has been associated with a more favourable metabolic outcome (10). Whether this has led to improvement in survival rate is however unknown. Data on mortality in patients with AI are discordant (6, 11-14). Despite having GC deficiency in common, PAI and SAI have

For peer review only - http://bmjopen.bmj.com/site/about/guidelines.xhtml

different aetiologies, hence they are two different diseases. SAI is usually associated with other pituitary deficiencies such as growth hormone deficiency, which per se could lead to increased risk of cardiovascular diseases and death (15). On the other hand, PAI is often associated with other autoimmune diseases which may play an important role on patients' outcome (16).

No previous studies have so far synthesized the available evidence on mortality in patients with AI. In this systematic review, we will analyse overall and disease specific mortality, with focus on cardiovascular and infectious diseases in patients with PAI and SAI. In addition, we will analyse time trends in mortality to better understand whether optimization of treatment strategies for AI over time have improved survival.

Study aims and objectives

The primary aim of this systematic review is to identify, critically appraise and synthetize available data on all-cause mortality in adult patients with AI. Secondary aims include the assessment of cardiovascular mortality, mortality due to infectious diseases and time trends in mortality.

Methods and analysis

The protocol for this review follows the recommendation of the preferred reporting items for systematic review and meta-analysis protocols (PRISMA-P) statement and has been registered in PROSPERO with registration number: CRD42023416253 (17). In the final systematic review, we will describe any deviation from the protocol. The final systematic review will be reported according to the PRISMA guidelines (17).

Eligibility criteria

The population of interest will be adult patients with AI, and this includes patients with PAI (including CAH) and SAI. Studies reporting all-cause, cardiovascular and infectious diseases mortality in patients with AI, will be eligible for inclusion. Time trend analysis for mortality in patients with AI will be performed. Data across different age groups, gender, and ethnicity

For peer review only - http://bmjopen.bmj.com/site/about/guidelines.xhtml

will be analyzed, and there will be no exclusion by country or language. Studies not including a comparison group (i.e. a background population) will be excluded. In addition, review articles, non-peer reviewed articles, commentaries, proceedings, laboratory science studies, case studies, case series, and other studies that do not allow the calculation of rates of the outcomes will be excluded from this systematic review. If an eligible study does not report the incidence of the prespecified outcomes, we will attempt to contact the study authors to request for the respective details.

Information sources

Our data sources will include electronic databases, conference abstracts, gray literature, and researchers and authors themselves. A systematic search will be performed through Medline, Embase, Cochrane and Web of Science using commonly used phrases stated in related literature, in addition to controlled vocabulary, i.e. Medical Subject Headings (MeSH) terms in Ovid Medline and Cochrane Central, and Emtree terms in Embase. The databases will be searched from their inception date until the end of May 2023. We will review reference lists and citations of included studies in order to find additional relevant articles. In addition, we will perform an updated search at the completion of the systematic review to ensure that studies published after the first search will be included.

Search strategy

Our initial search syntax for Ovid Medline is given below and this will be adapted in searching the other databases.

1 Adrenal Insufficiency/ or Addison Disease/ or Hypoadrenocorticism, Familial/ or Adrenoleukodystrophy/ or Hypoaldosteronism/ or Waterhouse-Friedrichsen syndrome/ or Hypopituitarism/ or Adrenal Hyperplasia, Congenital/

2 ('Adrenal Insufficiency' or Hypoadrenalism or 'Adrenal Gland Hypofunction' or Addison* or 'Adrenocortical Insufficiency' or 'Familial Hypoadrenocorticism' or

For peer review only - http://bmjopen.bmj.com/site/about/guidelines.xhtml

For peer review only

Adrenoleukodystrophy or Hypoaldosteronism or 'Waterhouse-Friedrichsen syndrome' or 'Complex Glycerol Kinase Deficiency' or 'Xp21 Contiguous Gene Deletion Syndrome' or 'X-linked Adrenal Hypoplasia' or 'X-Linked Addison Disease' or 'Cytomegalic Adrenocortical Hypoplasia' or 'Congenital Adrenal Hypoplasia' or 'Type IV Renal Tubular Acidosis' or 'Purpura Fulminans' or 'Meningococcal Hemorrhagic Adrenalitis' or Hypopituitarism or 'Congenital Adrenal Hyperplasia').tw,kf.

3 (mortality or death* or ((fatal or long-term) adj2 outcome*)).tw,kf. /freq=2

4 Mortality/ or Death/ or Survival/ or Fatal Outcome/ or Mortality.fs.

5 (exp animal/ or exp invertebrate/ or animal experiment/ or animal model/ or exp plant/ or exp fungus/) not exp human/

6 1 or 2

7 3 or 4

8 6 and 7

9 8 not 5

Selection process

Articles will be independently screened by two reviewers using the Rayyan web application for systematic reviews. First, articles will be screened by titles and/or abstracts. The screened articles will be classified into three groups: relevant, irrelevant, and uncertain. Articles that both reviewers consider to be irrelevant will be excluded from the review. For articles that are considered relevant or uncertain based on their title and/or abstract, the full text article will be retrieved to assess eligibility. Potentially relevant articles selected by at least one author will be investigated and evaluated in full. Next, the full text of the eligible articles will be reviewed and a list of articles to be included by each reviewer will be created. The two lists will then be compared, and the discrepancy will be discussed. If no agreement is reached, the

For peer review only

For peer review only - http://bmjopen.bmj.com/site/about/guidelines.xhtml

entire team will make the final decision. A flow diagram showing the selection process will be created using the PRISMA guidelines.

Data extraction

Predefined information of interest from included studies will be extracted using a data extraction form. This will help standardize the information recorded and aid analyses. The following data will be extracted independently by two reviewers: study name (along with the name of the first author and year of publication), country where the study was conducted, study size, source from which patients or study participants were selected, study design, disease duration, data on GC treatment, outcomes definition, age, gender, ethnicity, incidence or prevalence with 95% confidence intervals (CIs) or the number of patients achieving the outcomes. If the required data are inadequate, unclear, or missing from the article, authors will be contacted. If needed, missing information will be calculated from the available data when possible. In case of studies with overlapping population, only the study with the largest sample size and higher quality will be included. The data extraction will be discussed by two reviewers, and the entire team will arbitrate in the case of any disagreement.

Quality assessment

The quality of the studies will be independently assessed by two authors using the Newcastle–Ottawa quality assessment scale (NOS). Critical appraisal of articles will be performed to assess the methodological quality of each article. The quality appraisal will be undertaken by two reviewers and a third reviewer will arbitrate in the case of any disagreement.

Data synthesis

Characteristics and main results of the included studies will be presented in descriptive tables and the gathered evidence will be synthesized narratively.

For peer review only - http://bmjopen.bmj.com/site/about/guidelines.xhtml

We will calculate rate of mortality in each study if possible. If the included studies are sufficiently homogeneous, we will undertake a meta-analysis by pooling estimates across studies using the random effects approach.

Heterogeneity between studies will be assessed using the Higgins I^2 statistic (18). I^2 values of 0–30% will be considered as minimal heterogeneity, 31–50% moderate heterogeneity, and > 50% substantial heterogeneity (18). As PAI and SAI are two different entities, we will conduct separate subanalyses on mortality in PAI and SAI. If data are sufficient, we will conduct subgroup analysis to explore potential reasons for heterogeneity based on the quality of study, country, age, gender, and ethnicity. In addition, to analyze time trends in mortality in AI patients a subgroup analysis will be performed based on the time during which the studies were conducted. Begg's funnel plot and Egger's test will be used to assess publication bias (19). The former is a scatter plot of the log odds ratios (ORs) of individual studies on the x-axis against 1/ standard error (SE) of each study on the y-axis(19). Egger's test is a linear regression test of the normalized effect estimate (log OR/SE) against its precision (1/SE) (19). P value of < 0.10 on Egger's test or an asymmetrical funnel plot will be considered to indicate the presence of publication bias.

Ethical issues

This systematic review does not require ethical approval or informed consent because it will be based only on previously published data and does not implicate any direct contact with individual patients.

Discussion

Adrenal insufficiency is a life-threatening disorder. In the past, most patients with AI died within a few years after diagnosis due to scarce treatment options (3). Thanks to the discovery of cortisone in 1940s, the survival of patients with AI has improved (3). In the 1960s, Mason et al. showed that PAI patients on GC replacement therapy had a mortality rate similar to that of the background population (20). In line with that study, a recent Norwegian study, including 811 patients with PAI, showed no difference in mortality rate between PAI patients and the

For peer review only - http://bmjopen.bmj.com/site/about/guidelines.xhtml

general Norwegian population (11). Conversely, our group showed that mortality in PAI patients was 2-fold increased in comparison with the general Swedish population, after following 1675 patients with PAI between 1987 and 2001 (6). More recent data from UK have confirmed this finding showing a more than 2-fold excess mortality in patients with PAI (16). Mortality in AI patients has been related to cardiovascular diseases, infections, acute adrenal crises, and malignancies (7). In a large retrospective cohort study including 6821 patients with AI from United Kingdom, Ngaosuwan et al. have recently shown that overall mortality is higher by approximately 80% in PAI and 50% in SAI in comparison with matched controls (7). Another study on the same cohort has showed that mortality due to cardiovascular disease was increased in both PAI and SAI compared with matched controls (21). Interestingly, patients with SAI had an increased risk of cerebrovascular events when compared with PAI patients (21).

Other common causes of death in AI are infectious diseases (7). A multicenter study including 2034 patients with PAI and SAI from the European Adrenal Insufficiency Registry (EU-AIR) showed that the major causes of death were cardiovascular disease (35%) and infectious diseases (15%)(22). In a population-based study, including 3299 patients with PAI, Bensing *et al.* have found that the risk of death due to infections was 6-fold increased compared to the general population (5). Moreover, in a cohort study of 1286 hypopituitary patients, Burman *et al.* have found that mortality from infections was increased in ACTH-deficient patients but not in patients without ACTH deficiency (12).

Inappropriate replacement therapy with glucocorticoids has been described as the main determinant of a poor prognosis in AI patients (23). Excessive cortisol exposure and glucocorticoid replacement therapy that does not mimic cortisol's circadian rhythm are indeed associated with the adverse metabolic profile, increased morbidity, and mortality (23, 24).

Over the last two decades, efforts have been made to optimize GC treatment, trying to reduce the total daily GC dose and optimise intercurrent illness management (9). In addition, novel treatment strategies have been introduced to better mimic the physiological diurnal variation in cortisol levels (10). Whether this has led to an improvement in long-term outcomes remains unknown. Previous studies conducted on this topic have shown controversial findings due to different study designs, populations and settings (5, 6, 11). Since AI is a rare disease, no single center has accumulated sufficient experience to draw definitive conclusions. Therefore, a

For peer review only - http://bmjopen.bmj.com/site/about/guidelines.xhtml

systematic review is needed to provide clearer insights into this topic. Increasing knowledge of AI mortality can lead to optimized management of AI and its comorbidities.

Contributions

DE conceived this study. FA, KD, DP and DE developed the study protocol. All the authors revised the study protocol. FA and KD will implement the systematic review under the supervision of DE, EB, RB, DC, BN, DP and GJ. EH will develop the search strings and conduct the study search. EB and BN will provide the statistical analysis expertise. EB, BN, and DE will conduct data analysis. FA and KD will perform the screening and extraction of data whereas DE, EB, RB, DC, BN and GJ will review the work.

Funding statement

This work received support from the Swedish state under the agreement between the Swedish government and the county councils, the ALF-agreement (ALFGBG-874631 and ALFGBG-960884).

Conflicts of interest and financial disclosure

None declared

For peer review only

References

1. Charmandari E, Nicolaides NC, Chrousos GP. Adrenal insufficiency. Lancet. 2014;383(9935):2152-67.
2. Husebye ES, Pearce SH, Krone NP, Kampe O. Adrenal insufficiency. Lancet. 2021;397(10274):613-29.
3. Kumar R, Wassif WS. Adrenal insufficiency. J Clin Pathol. 2022;75(7):435-42.
4. Dunlop D. Eighty-Six Cases of Addison's Disease. Br Med J. 1963;2(5362):887-91.
5. Bensing S, Brandt L, Tabaroj F, Sjoberg O, Nilsson B, Ekbom A, et al. Increased death risk and altered cancer incidence pattern in patients with isolated or combined autoimmune primary adrenocortical insufficiency. Clin Endocrinol (Oxf). 2008;69(5):697-704.
6. Bergthorsdottir R, Leonsson-Zachrisson M, Oden A, Johannsson G. Premature mortality in patients with Addison's disease: a population-based study. J Clin Endocrinol Metab. 2006;91(12):4849-53.
7. Ngaosuwan K, Johnston DG, Godsland IF, Cox J, Majeed A, Quint JK, et al. Increased Mortality Risk in Patients With Primary and Secondary Adrenal Insufficiency. J Clin Endocrinol Metab. 2021;106(7):e2759-e68.
8. Ngaosuwan K, Johnston DG, Godsland IF, Cox J, Majeed A, Quint JK, et al. Mortality Risk in Patients With Adrenal Insufficiency Using Prednisolone or Hydrocortisone: A Retrospective Cohort Study. J Clin Endocrinol Metab. 2021;106(8):2242-51.
9. Johannsson G, Skrtic S, Lennernas H, Quinkler M, Stewart PM. Improving outcomes in patients with adrenal insufficiency: a review of current and future treatments. Curr Med Res Opin. 2014;30(9):1833-47.
10. Johannsson G, Nilsson AG, Bergthorsdottir R, Burman P, Dahlqvist P, Ekman B, et al. Improved cortisol exposure-time profile and outcome in patients with adrenal insufficiency: a prospective randomized trial of a novel hydrocortisone dual-release formulation. J Clin Endocrinol Metab. 2012;97(2):473-81.
11. Erichsen MM, Lovas K, Fougner KJ, Svartberg J, Hauge ER, Bollerslev J, et al. Normal overall mortality rate in Addison's disease, but young patients are at risk of premature death. Eur J Endocrinol. 2009;160(2):233-7.
12. Burman P, Mattsson AF, Johannsson G, Hoybye C, Holmer H, Dahlqvist P, et al. Deaths among adult patients with hypopituitarism: hypocortisolism during acute stress, and de novo malignant brain tumors contribute to an increased mortality. J Clin Endocrinol Metab. 2013;98(4):1466-75.
13. O'Reilly MW, Reulen RC, Gupta S, Thompson CA, Dineen R, Goulden EL, et al. ACTH and gonadotropin deficiencies predict mortality in patients treated for nonfunctioning pituitary adenoma: long-term follow-up of 519 patients in two large European centres. Clin Endocrinol (Oxf). 2016;85(5):748-56.
14. Hammarstrand C, Ragnarsson O, Hallen T, Andersson E, Skoglund T, Nilsson AG, et al. Higher glucocorticoid replacement doses are associated with increased mortality in patients with pituitary adenoma. Eur J Endocrinol. 2017;177(3):251-6.
15. Gola M, Bonadonna S, Doga M, Giustina A. Clinical review: Growth hormone and cardiovascular risk factors. J Clin Endocrinol Metab. 2005;90(3):1864-70.
16. Conrad N, Verbeke G, Molenberghs G, Goetschalckx L, Callender T, Cambridge G, et al. Autoimmune diseases and cardiovascular risk: a population-based study on 19 autoimmune diseases and 12 cardiovascular diseases in 22 million individuals in the UK. Lancet. 2022;400(10354):733-43.

For peer review only

For peer review only - http://bmjopen.bmj.com/site/about/guidelines.xhtml

17. Shamseer L, Moher D, Clarke M, Ghersi D, Liberati A, Petticrew M, et al. Preferred reporting items for systematic review and meta-analysis protocols (PRISMA-P) 2015: elaboration and explanation. BMJ. 2015;350:g7647.

18. Higgins JP, Thompson SG. Quantifying heterogeneity in a meta-analysis. Stat Med. 2002;21(11):1539-58.

19. Egger M, Smith GD. Bias in location and selection of studies. BMJ. 1998;316(7124):61-6.

20. Mason AS, Meade TW, Lee JA, Morris JN. Epidemiological and clinical picture of Addison's disease. Lancet. 1968;2(7571):744-7.

21. Ngaosuwan K, Johnston DG, Godsland IF, Cox J, Majeed A, Quint JK, et al. Cardiovascular Disease in Patients With Primary and Secondary Adrenal Insufficiency and the Role of Comorbidities. J Clin Endocrinol Metab. 2021;106(5):1284-93.

22. Quinkler M, Ekman B, Zhang P, Isidori AM, Murray RD, Investigators E-A. Mortality data from the European Adrenal Insufficiency Registry-Patient characterization and associations. Clin Endocrinol (Oxf). 2018;89(1):30-5.

23. Rahvar AH, Haas CS, Danneberg S, Harbeck B. Increased Cardiovascular Risk in Patients with Adrenal Insufficiency: A Short Review. Biomed Res Int. 2017;2017:3691913.

24. Esposito D, Bobbio E, Di Fraia R, Mone P, Accardo G, De Bellis A, et al. Patients with adrenal insufficiency have cardiovascular features associated with hypovolemia. Endocrine. 2020;70(2):412-20.

For peer review only

For peer review only - http://bmjopen.bmj.com/site/about/guidelines.xhtml

PRISMA-P 2015 Checklist

This checklist has been adapted for use with protocol submissions to *Systematic Reviews* from Table 3 in Moher D et al: Preferred reporting items for systematic review and meta-analysis protocols (PRISMA-P) 2015 statement. *Systematic Reviews* 2015 **4**:1

Section/topic	#	Checklist item	Information reported: Yes	Information reported: No	Line number(s)
**ADMINISTRATIVE INFORMATION**					
**Title**					
Identification	1a	Identify the report as a protocol of a systematic review	X		
Update	1b	If the protocol is for an update of a previous systematic review, identify as such		X	
**Registration**	2	If registered, provide the name of the registry (e.g., PROSPERO) and registration number in the Abstract	X		
**Authors**					
Contact	3a	Provide name, institutional affiliation, and e-mail address of all protocol authors; provide physical mailing address of corresponding author	X		
Contributions	3b	Describe contributions of protocol authors and identify the guarantor of the review	X		
**Amendments**	4	If the protocol represents an amendment of a previously completed or published protocol, identify as such and list changes; otherwise, state plan for documenting important protocol amendments		X	
**Support**					
Sources	5a	Indicate sources of financial or other support for the review	X		
Sponsor	5b	Provide name for the review funder and/or sponsor	X		
Role of sponsor/funder	5c	Describe roles of funder(s), sponsor(s), and/or institution(s), if any, in developing the protocol		X	
**INTRODUCTION**					
**Rationale**	6	Describe the rationale for the review in the context of what is already known	X		
**Objectives**	7	Provide an explicit statement of the question(s) the review will address with reference to participants, interventions, comparators, and outcomes (PICO)	X		
**METHODS**					

BioMed Central
The Open Access Publisher

For peer review only - http://bmjopen.bmj.com/site/about/guidelines.xhtml

Section/topic	#	Checklist item	Information reported Yes	No	Line number(s)
**Eligibility criteria**	8	Specify the study characteristics (e.g., PICO, study design, setting, time frame) and report characteristics (e.g., years considered, language, publication status) to be used as criteria for eligibility for the review	X		
**Information sources**	9	Describe all intended information sources (e.g., electronic databases, contact with study authors, trial registers, or other grey literature sources) with planned dates of coverage	X		
**Search strategy**	10	Present draft of search strategy to be used for at least one electronic database, including planned limits, such that it could be repeated		X	
STUDY RECORDS					
Data management	11a	Describe the mechanism(s) that will be used to manage records and data throughout the review	X		
Selection process	11b	State the process that will be used for selecting studies (e.g., two independent reviewers) through each phase of the review (i.e., screening, eligibility, and inclusion in meta-analysis)	X		
Data collection process	11c	Describe planned method of extracting data from reports (e.g., piloting forms, done independently, in duplicate), any processes for obtaining and confirming data from investigators	X		
**Data items**	12	List and define all variables for which data will be sought (e.g., PICO items, funding sources), any pre-planned data assumptions and simplifications	X		
**Outcomes and prioritization**	13	List and define all outcomes for which data will be sought, including prioritization of main and additional outcomes, with rationale	X		
**Risk of bias in individual studies**	14	Describe anticipated methods for assessing risk of bias of individual studies, including whether this will be done at the outcome or study level, or both; state how this information will be used in data synthesis	X		
DATA					
**Synthesis**	15a	Describe criteria under which study data will be quantitatively synthesized	X		
	15b	If data are appropriate for quantitative synthesis, describe planned summary measures, methods of handling data, and methods of combining data from studies, including any planned exploration of consistency (e.g., I^2, Kendall's tau)	X		
	15c	Describe any proposed additional analyses (e.g., sensitivity or subgroup analyses, meta-regression)	X		
	15d	If quantitative synthesis is not appropriate, describe the type of summary planned		X	
**Meta-bias(es)**	16	Specify any planned assessment of meta-bias(es) (e.g., publication bias across studies, selective reporting within studies)	X		
**Confidence in cumulative evidence**	17	Describe how the strength of the body of evidence will be assessed (e.g., GRADE)	X		

For peer review only

BioMed Central
The Open Access Publisher

BMJ Open

Mortality in patients with adrenal insufficiency: a protocol for a systematic review and meta-analysis

Journal:	*BMJ Open*
Manuscript ID	bmjopen-2023-076582.R1
Article Type:	Protocol
Date Submitted by the Author:	15-Nov-2023
Complete List of Authors:	Allosso, Francesca; University of Campania Luigi Vanvitelli, Department of Advanced Medical and Surgical Sciences<br>Dalakas, Konstantinos; University of Gothenburg, Department of Internal Medicine and Clinical Nutrition; Sahlgrenska University Hospital, Department of Endocrinology<br>Bergthorsdottir, Ragnhildur; University of Gothenburg, Department of Internal Medicine and Clinical Nutrition; Sahlgrenska University Hospital, Department of Endocrinology<br>Chantzichristos, Dimitrios; University of Gothenburg, Department of Internal Medicine and Clinical Nutrition; Sahlgrenska University Hospital, Department of Endocrinology<br>Hessman, Eva; University of Gothenburg, Biomedical Library, Gothenburg University Library<br>Nwaru, Bright; University of Gothenburg Institute of Medicine, Krefting Research Centre; University of Gothenburg Wallenberg Centre for Molecular and Translational Medicine<br>Bobbio, Emanuele; Sahlgrenska University Hospital, Department of Cardiology; University of Gothenburg,<br>Pasquali, Daniela; University of Campania Luigi Vanvitelli, Department of Advanced Medical and Surgical Sciences<br>Johannsson, Gudmundur; University of Gothenburg, Department of Internal Medicine and Clinical Nutrition; Sahlgrenska University Hospital, Department of Endocrinology<br>Esposito, Daniela; University of Gothenburg, Department of Internal Medicine and Clinical Nutrition, Institute of Medicine, Sahlgrenska Academy; Sahlgrenska University Hospital, Department of Endocrinology
**Primary Subject Heading**:	Diabetes and endocrinology
Secondary Subject Heading:	Epidemiology
Keywords:	Pituitary disorders < DIABETES & ENDOCRINOLOGY, General endocrinology < DIABETES & ENDOCRINOLOGY, CARDIOLOGY, Adrenal disorders < DIABETES & ENDOCRINOLOGY, Mortality

SCHOLARONE™
Manuscripts

Mortality in patients with adrenal insufficiency: a protocol for a systematic review and meta-analysis

Francesca Allosso*[1], Konstantinos Dalakas*[2,3], Ragnhildur Bergthorsdottir[2,3], Dimitrios Chantzichristos[2,3], Eva Hessman[4], Bright I. Nwaru[5,6], Emanuele Bobbio[7,8], Daniela Pasquali[1], Gudmundur Johannsson[2,3], Daniela Esposito[2,3]

*These authors contributed equally

Affiliation:

[1]Department of Advanced Medical and Surgical Sciences, University of Campania "Luigi Vanvitelli", Naples, Italy; [2] Department of Internal Medicine and Clinical Nutrition, Institute of Medicine, Sahlgrenska Academy, University of Gothenburg, Gothenburg, Sweden; [3]Department of Endocrinology, Sahlgrenska University Hospital, Gothenburg, Sweden; [4]Biomedical Library, Gothenburg University Library, University of Gothenburg, Gothenburg, Sweden; [5] Krefting Research Centre, Institute of Medicine, Sahlgrenska Academy, University of Gothenburg, Gothenburg, Sweden; [6] Wallenberg Centre for Molecular and Translational Medicine, Institute of Medicine, University of Gothenburg, Gothenburg, Sweden; [7]Department of Cardiology, Sahlgrenska University Hospital, Gothenburg, Sweden; [8]Institute of Medicine at Sahlgrenska Academy, University of Gothenburg, Gothenburg, Sweden

Key Words: adrenal insufficiency, hypopituitarism, mortality, glucocorticoid replacement

Abstract: 230 words

Manuscript: 2375 words

References: 24

Running title: Mortality in adrenal insufficiency

For peer review only

For peer review only - http://bmjopen.bmj.com/site/about/guidelines.xhtml

Corresponding author and person to whom reprint requests should be addressed:
Daniela Esposito, MD, PhD, Department of Endocrinology, Sahlgrenska University Hospital Institute of Medicine, Sahlgrenska Academy, University of Gothenburg, Gothenburg, Sweden. Email: daniela.esposito@gu.se

For peer review only

For peer review only - http://bmjopen.bmj.com/site/about/guidelines.xhtml

Abstract

Introduction

Adrenal insufficiency (AI) is a rare disorder characterized by an impaired secretion of glucocorticoids from the adrenal glands. Treatment strategies for AI have developed over time with reduced glucocorticoid replacement doses and improved circadian exposure regimens, but whether this has resulted in better survival is unknown.

The main purpose of this systematic review is to gather and synthesize available evidence on long-term mortality in patients with AI. Secondary aim is to study causes of death, with focus on cardiovascular and infectious diseases, and time trends in mortality in AI patients.

Methods and analysis

Studies published from the inception of respective databases (Medline, Embase, Cochrane and Web of Science) until the end of May 2023 will be systematically synthetized. Observational studies with a reference population will be included, and their quality will be assessed using the Newcastle-Ottawa scale. Data collected will be narratively integrated and a meta-analysis will be performed to pool data from studies considered homogenous. The systematic review will be reported following the PRISMA guidelines. This will be the first systematic review assessing mortality, causes of death and time trends in mortality in AI patients. The findings of this systematic review will be of value for both patients and healthcare providers.

Ethics and dissemination

This systematic review does not require ethical approval or informed consent because it will be based on previously published data only and does not implicate any direct contact with individual patients.

PROSPERO registration number: CRD42023416253

For peer review only

For peer review only - http://bmjopen.bmj.com/site/about/guidelines.xhtml

Strengths and limitations of this study

- This systematic review will for the first time provide a comprehensive synthesis of overall mortality and time trends in mortality in patients with adrenal insufficiency.
- The protocol of this systematic review follows the Preferred Reporting Items for Systematic Review and Meta-Analysis Protocols guidelines.
- A detailed description of the review processes is presented in this protocol increasing the reproducibility of the systematic review.
- All available studies on mortality in adrenal insufficiency will be gathered from publicly available literature databases, without language or geographical limitations.
- A potential limitation can be high heterogeneity in the population, as both primary and secondary adrenal insufficiency are going to be included. However, separate sub-group analyses will be performed.

For peer review only

For peer review only - http://bmjopen.bmj.com/site/about/guidelines.xhtml

Introduction

Adrenal insufficiency (AI) is a rare disorder characterized by an impaired secretion of glucocorticoids from the adrenal cortex (1). Primary AI (PAI), with an estimated prevalence of 93-144/million in Europe, is caused by disorders of the adrenal glands and is usually associated with mineralocorticoid and androgen deficiency (1). It can be acquired or inherited as in congenital adrenal hyperplasia (CAH), usually due to steroid 21-hydroxylase deficiency (2). In western countries acquired PAI is most commonly due to an autoimmune-mediated adrenalitis, which can be isolated or combined with other autoimmune diseases (e.g. autoimmune polyglandular syndrome type 1 or 2) (2, 3). Other causes of PAI are tuberculosis and infectious diseases (3). Secondary AI (SAI), with an estimated prevalence of 150-280/million, is caused by adrenocorticotropic hormone (ACTH) deficiency secondary to a pituitary disease and is often associated with other pituitary hormone deficiencies (1, 2).

Treatment of AI consists of replacement therapy with glucocorticoids (GC) (1). In PAI, mineralocorticoid replacement therapy is also required while in SAI additional replacement therapy with thyroid hormone, sex steroids and growth hormone may be needed due to failure of other pituitary hormone axes (2). Before the introduction of GC therapy, most patients with PAI died within 2 years of diagnosis (4). Although long-term outcomes have markedly improved, mortality remains increased, with cardiovascular disease being the leading cause of death (5-7).

Standard replacement treatments in AI include short-acting glucocorticoids such as hydrocortisone and cortisone acetate, usually given 2-3 times daily (8). In some countries other GCs such as prednisolone are used (2). However, conventional GC replacement fails to restore a circadian cortisol profile in patients with AI.

There have been attempts to improve the dosing regimen of GC replacement therapy and the management of intercurrent illnesses (9). The introduction of new therapies, as once-daily modified release hydrocortisone that better mimics the endogen circadian production of cortisol, has been associated with a more favourable metabolic outcome (10). Whether this has led to improvement in survival rate is however unknown. Data on mortality in patients with AI are discordant (6, 11-14). Despite having GC deficiency in common, PAI and SAI have

For peer review only - http://bmjopen.bmj.com/site/about/guidelines.xhtml

different aetiologies. SAI is usually associated with other pituitary deficiencies such as growth hormone deficiency, which per se could lead to increased risk of cardiovascular diseases and death (15). On the other hand, PAI is often associated with other autoimmune diseases which may play an important role on patients' outcome (16).

No previous studies have so far synthesized the available evidence on mortality in patients with AI. In this systematic review, we will analyse overall and disease specific mortality in patients with PAI and SAI. In addition, we will analyse time trends in mortality to better understand whether optimization of treatment strategies for AI over time have improved survival.

Study aims and objectives

The primary aim of this systematic review is to identify, critically appraise and synthetize available data on long-term mortality in adult patients with AI. Secondary aims include the assessment of cardiovascular mortality and time trends in mortality.

Methods and analysis

The protocol for this review follows the recommendation of the preferred reporting items for systematic review and meta-analysis protocols (PRISMA-P) statement and has been registered in PROSPERO with registration number: CRD42023416253 (17).

This systematic review was planned in November 2022 and is expected to be finalized in June 2024. In the final systematic review, we will describe any deviation from the protocol. The final systematic review will be reported according to the PRISMA guidelines (17).

Eligibility criteria

The population of interest will be adult patients with AI, and this includes patients with PAI (including CAH) and SAI. Studies reporting long-term mortality (defined as death occurring ≥ 1 year from the diagnosis of AI) will be eligible for inclusion. Time trend analysis for mortality in patients with AI will be performed. Data across different age groups, gender, and ethnicity will be analyzed, and there will be no exclusion by country or language. Patients with

For peer review only - http://bmjopen.bmj.com/site/about/guidelines.xhtml

Waterhouse-Friderichsen syndrome, leukodystrophy and Type 4 Renal tubular acidosis will be excluded from the analysis. Studies not including a comparison group (i.e. a background population) will be excluded. In addition, review articles, non-peer reviewed articles, commentaries, proceedings, laboratory science studies, case studies, case series, and other studies that do not allow the calculation of rates of the outcomes will be excluded from this systematic review. If an eligible study does not report the incidence of the prespecified outcomes, we will attempt to contact the study authors to request for the respective details.

Information sources

Our data sources will include electronic databases, conference abstracts, gray literature, and researchers and authors themselves. A systematic search will be performed through Medline, Embase, Cochrane and Web of Science using commonly used phrases stated in related literature, in addition to controlled vocabulary, i.e. Medical Subject Headings (MeSH) terms in Ovid Medline and Cochrane Central, and Emtree terms in Embase. The databases will be searched from their inception date until the end of May 2023. Broad and inclusive search terms will be used in order to limit the risk of omitting any relevant studies.

We will review reference lists and citations of included studies in order to find additional relevant articles. In addition, we will perform an updated search at the completion of the systematic review to ensure that studies published after the first search will be included.

Search strategy

Our initial search syntax for Ovid Medline is given below and this will be adapted in searching the other databases.

1 Adrenal Insufficiency/ or Addison Disease/ or Hypoadrenocorticism, Familial/ or Adrenoleukodystrophy/ or Hypoaldosteronism/ or Waterhouse-Friedrichsen syndrome/ or Hypopituitarism/ or Adrenal Hyperplasia, Congenital/

For peer review only

2	('Adrenal Insufficiency' or Hypoadrenalism or 'Adrenal Gland Hypofunction' or Addison* or 'Adrenocortical Insufficiency' or 'Familial Hypoadrenocorticism' or Adrenoleukodystrophy or Hypoaldosteronism or 'Waterhouse-Friedrichsen syndrome' or 'Complex Glycerol Kinase Deficiency' or 'Xp21 Contiguous Gene Deletion Syndrome' or 'X-linked Adrenal Hypoplasia' or 'X-Linked Addison Disease' or 'Cytomegalic Adrenocortical Hypoplasia' or 'Congenital Adrenal Hypoplasia' or 'Type IV Renal Tubular Acidosis' or 'Purpura Fulminans' or 'Meningococcal Hemorrhagic Adrenalitis' or Hypopituitarism or 'Congenital Adrenal Hyperplasia').tw,kf.
3	(mortality or death* or ((fatal or long-term) adj2 outcome*)).tw,kf. /freq=2
4	Mortality/ or Death/ or Survival/ or Fatal Outcome/ or Mortality.fs.
5	(exp animal/ or exp invertebrate/ or animal experiment/ or animal model/ or exp plant/ or exp fungus/) not exp human/
6	1 or 2
7	3 or 4
8	6 and 7
9	8 not 5

For peer review only

Selection process

Articles will be independently screened by two reviewers using the Rayyan web application for systematic reviews. First, articles will be screened by titles and/or abstracts. The screened articles will be classified into three groups: relevant, irrelevant, and uncertain. Articles that both reviewers consider to be irrelevant will be excluded from the review. For articles that are considered relevant or uncertain based on their title and/or abstract, the full text article will be retrieved to assess eligibility. Potentially relevant articles selected by at least one author will be investigated and evaluated in full. Next, the full text of the eligible articles will

For peer review only - http://bmjopen.bmj.com/site/about/guidelines.xhtml

be reviewed and a list of articles to be included by each reviewer will be created. The two lists will then be compared, and the discrepancy will be discussed. If no agreement is reached, the entire team will make the final decision. A flow diagram showing the selection process will be created using the PRISMA guidelines.

Data extraction

Predefined information of interest from included studies will be extracted using a data extraction form. This will help standardize the information recorded and aid analyses. The following data will be extracted independently by two reviewers: study name (along with the name of the first author and year of publication), country where the study was conducted, study size, source from which patients or study participants were selected, study design, definition of AI, definition of CAH, disease duration, outcomes definition, age, gender, ethnicity, incidence or prevalence with 95% confidence intervals (CIs) or the number of patients achieving the outcomes. Data on causes of PAI and SAI will be systematically gathered, as underlying conditions themselves may independently influence the outcomes. Data on doses of CG and MC will be collected, as treatment strategies have changed over time. Studies including patients with hypopituitarism will be included with the aim to gather data on patients with SAI. If the required data are inadequate, unclear, or missing from the article, authors will be contacted. If needed, missing information will be calculated from the available data when possible. In case of studies with overlapping population, only the study with the largest sample size and higher quality will be included. The data extraction will be discussed by two reviewers, and the entire team will arbitrate in the case of any disagreement.

Quality assessment

The quality of the studies will be independently assessed by two authors using the Newcastle–Ottawa quality assessment scale (NOS). Critical appraisal of articles will be performed to assess the methodological quality of each article. The quality appraisal will be undertaken by two reviewers and a third reviewer will arbitrate in the case of any disagreement.

For peer review only - http://bmjopen.bmj.com/site/about/guidelines.xhtml

Data synthesis

Characteristics and main results of the included studies will be presented in descriptive tables and the gathered evidence will be synthesized narratively.

We will calculate rate of mortality in each study if possible. If the included studies are sufficiently homogeneous, we will undertake a meta-analysis by pooling estimates across studies using the random effects approach.

Heterogeneity between studies will be assessed using the Higgins I^2 statistic (18). I^2 values of 0–30% will be considered as minimal heterogeneity, 31–50% moderate heterogeneity, and > 50% substantial heterogeneity (18). As PAI and SAI are two different entities, we will conduct separate subanalyses on mortality in PAI and SAI. If data are sufficient, we will conduct subgroup analysis to explore potential reasons for heterogeneity based on the quality of study, country, age, gender, and ethnicity. A subgroup analysis will be also conduct for patients with CAH. In addition, to analyze time trends in mortality in AI patients, a subgroup analysis will be performed based on the study period of the included studies. Specifically, we will collect detailed data on time period of inclusion of patients with AI and reference population. Studies that analyzed mortality based on reference population not living in the same time period as patients with AI will be excluded.

Begg's funnel plot and Egger's test will be used to assess publication bias (19). The former is a scatter plot of the log odds ratios (ORs) of individual studies on the x-axis against 1/ standard error (SE) of each study on the y-axis(19). Egger's test is a linear regression test of the normalized effect estimate (log OR/SE) against its precision (1/SE) (19). P value of < 0.10 on Egger's test or an asymmetrical funnel plot will be considered to indicate the presence of publication bias.

Ethical issues

This systematic review does not require ethical approval or informed consent because it will be based only on previously published data and does not implicate any direct contact with individual patients.

For peer review only - http://bmjopen.bmj.com/site/about/guidelines.xhtml

Patient and public involvement

No patient will be involved in this systematic review

Discussion

Adrenal insufficiency is a life-threatening disorder. In the past, most patients with AI died within a few years after diagnosis due to scarce treatment options (3). Thanks to the discovery of cortisone in 1940s, the survival of patients with AI has improved (3). In the 1960s, Mason et al. showed that PAI patients on GC replacement therapy had a mortality rate similar to that of the background population (20). In line with that study, a recent Norwegian study, including 811 patients with PAI, showed no difference in mortality rate between PAI patients and the general Norwegian population (11). Conversely, our group showed that mortality in PAI patients was 2-fold increased in comparison with the general Swedish population, after following 1675 patients with PAI between 1987 and 2001 (6). More recent data from UK have confirmed this finding showing a more than 2-fold excess mortality in patients with PAI (16). Mortality in AI patients has been related to cardiovascular diseases, infections, acute adrenal crises, and malignancies (7). In a large retrospective cohort study including 6821 patients with AI from United Kingdom, Ngaosuwan et al. have recently shown that overall mortality is higher by approximately 80% in PAI and 50% in SAI in comparison with matched controls (7). Another study on the same cohort has showed that mortality due to cardiovascular disease was increased in both PAI and SAI compared with matched controls (21). Interestingly, patients with SAI had an increased risk of cerebrovascular events when compared with PAI patients (21).

Other common causes of death in AI are infectious diseases (7). A multicenter study including 2034 patients with PAI and SAI from the European Adrenal Insufficiency Registry (EU-AIR) showed that the major causes of death were cardiovascular disease (35%) and infectious diseases (15%)(22). In a population-based study, including 3299 patients with PAI, Bensing *et al.* have found that the risk of death due to infections was 6-fold increased compared to the general population (5). Moreover, in a cohort study of 1286 hypopituitary patients, Burman

For peer review only - http://bmjopen.bmj.com/site/about/guidelines.xhtml

et al. have found that mortality from infections was increased in ACTH-deficient patients but not in patients without ACTH deficiency (12).

Inappropriate replacement therapy with GCs has been described as the main determinant of a poor prognosis in AI patients (23). Excessive cortisol exposure and glucocorticoid replacement therapy that does not mimic cortisol circadian rhythm are indeed associated with the adverse metabolic profile, increased morbidity, and mortality (23, 24).

Over the last two decades, efforts have been made to optimize GC treatment, trying to reduce the total daily GC dose and optimise intercurrent illness management (9). In addition, novel treatment strategies have been introduced to better mimic the physiological diurnal variation in cortisol levels (10). Whether this has led to an improvement in long-term outcomes remains unknown. Previous studies conducted on this topic have shown controversial findings due to different study designs, populations and settings (5, 6, 11). Since AI is a rare disease, no single center has accumulated sufficient experience to draw definitive conclusions. Therefore, a systematic review is needed to provide clearer insights into this topic. Increasing knowledge of AI mortality can lead to optimized management of AI and its comorbidities.

Contributions

DE conceived this study. FA, KD, DP and DE developed the study protocol. All the authors revised the study protocol. FA and KD will implement the systematic review under the supervision of DE, EB, RB, DC, BN, DP and GJ. EH will develop the search strings and conduct the study search. EB and BN will provide the statistical analysis expertise. EB, BN, and DE will conduct data analysis. FA and KD will perform the screening and extraction of data whereas DE, EB, RB, DC, BN and GJ will review the work.

Funding statement

This work received support from the Swedish state under the agreement between the Swedish government and the county councils, the ALF-agreement (ALFGBG-874631 and ALFGBG-960884).

Conflicts of interest and financial disclosure

For peer review only - http://bmjopen.bmj.com/site/about/guidelines.xhtml

None declared

For peer review only

For peer review only - http://bmjopen.bmj.com/site/about/guidelines.xhtml

References

1. Charmandari E, Nicolaides NC, Chrousos GP. Adrenal insufficiency. Lancet. 2014;383(9935):2152-67.
2. Husebye ES, Pearce SH, Krone NP, Kampe O. Adrenal insufficiency. Lancet. 2021;397(10274):613-29.
3. Kumar R, Wassif WS. Adrenal insufficiency. J Clin Pathol. 2022;75(7):435-42.
4. Dunlop D. Eighty-Six Cases of Addison's Disease. Br Med J. 1963;2(5362):887-91.
5. Bensing S, Brandt L, Tabaroj F, Sjoberg O, Nilsson B, Ekbom A, et al. Increased death risk and altered cancer incidence pattern in patients with isolated or combined autoimmune primary adrenocortical insufficiency. Clin Endocrinol (Oxf). 2008;69(5):697-704.
6. Bergthorsdottir R, Leonsson-Zachrisson M, Oden A, Johannsson G. Premature mortality in patients with Addison's disease: a population-based study. J Clin Endocrinol Metab. 2006;91(12):4849-53.
7. Ngaosuwan K, Johnston DG, Godsland IF, Cox J, Majeed A, Quint JK, et al. Increased Mortality Risk in Patients With Primary and Secondary Adrenal Insufficiency. J Clin Endocrinol Metab. 2021;106(7):e2759-e68.
8. Ngaosuwan K, Johnston DG, Godsland IF, Cox J, Majeed A, Quint JK, et al. Mortality Risk in Patients With Adrenal Insufficiency Using Prednisolone or Hydrocortisone: A Retrospective Cohort Study. J Clin Endocrinol Metab. 2021;106(8):2242-51.
9. Johannsson G, Skrtic S, Lennernas H, Quinkler M, Stewart PM. Improving outcomes in patients with adrenal insufficiency: a review of current and future treatments. Curr Med Res Opin. 2014;30(9):1833-47.
10. Johannsson G, Nilsson AG, Bergthorsdottir R, Burman P, Dahlqvist P, Ekman B, et al. Improved cortisol exposure-time profile and outcome in patients with adrenal insufficiency: a prospective randomized trial of a novel hydrocortisone dual-release formulation. J Clin Endocrinol Metab. 2012;97(2):473-81.
11. Erichsen MM, Lovas K, Fougner KJ, Svartberg J, Hauge ER, Bollerslev J, et al. Normal overall mortality rate in Addison's disease, but young patients are at risk of premature death. Eur J Endocrinol. 2009;160(2):233-7.
12. Burman P, Mattsson AF, Johannsson G, Hoybye C, Holmer H, Dahlqvist P, et al. Deaths among adult patients with hypopituitarism: hypocortisolism during acute stress, and de novo malignant brain tumors contribute to an increased mortality. J Clin Endocrinol Metab. 2013;98(4):1466-75.
13. O'Reilly MW, Reulen RC, Gupta S, Thompson CA, Dineen R, Goulden EL, et al. ACTH and gonadotropin deficiencies predict mortality in patients treated for nonfunctioning pituitary adenoma: long-term follow-up of 519 patients in two large European centres. Clin Endocrinol (Oxf). 2016;85(5):748-56.
14. Hammarstrand C, Ragnarsson O, Hallen T, Andersson E, Skoglund T, Nilsson AG, et al. Higher glucocorticoid replacement doses are associated with increased mortality in patients with pituitary adenoma. Eur J Endocrinol. 2017;177(3):251-6.
15. Gola M, Bonadonna S, Doga M, Giustina A. Clinical review: Growth hormone and cardiovascular risk factors. J Clin Endocrinol Metab. 2005;90(3):1864-70.
16. Conrad N, Verbeke G, Molenberghs G, Goetschalckx L, Callender T, Cambridge G, et al. Autoimmune diseases and cardiovascular risk: a population-based study on 19 autoimmune diseases and 12 cardiovascular diseases in 22 million individuals in the UK. Lancet. 2022;400(10354):733-43.

For peer review only - http://bmjopen.bmj.com/site/about/guidelines.xhtml

17. Shamseer L, Moher D, Clarke M, Ghersi D, Liberati A, Petticrew M, et al. Preferred reporting items for systematic review and meta-analysis protocols (PRISMA-P) 2015: elaboration and explanation. BMJ. 2015;350:g7647.

18. Higgins JP, Thompson SG. Quantifying heterogeneity in a meta-analysis. Stat Med. 2002;21(11):1539-58.

19. Egger M, Smith GD. Bias in location and selection of studies. BMJ. 1998;316(7124):61-6.

20. Mason AS, Meade TW, Lee JA, Morris JN. Epidemiological and clinical picture of Addison's disease. Lancet. 1968;2(7571):744-7.

21. Ngaosuwan K, Johnston DG, Godsland IF, Cox J, Majeed A, Quint JK, et al. Cardiovascular Disease in Patients With Primary and Secondary Adrenal Insufficiency and the Role of Comorbidities. J Clin Endocrinol Metab. 2021;106(5):1284-93.

22. Quinkler M, Ekman B, Zhang P, Isidori AM, Murray RD, Investigators E-A. Mortality data from the European Adrenal Insufficiency Registry-Patient characterization and associations. Clin Endocrinol (Oxf). 2018;89(1):30-5.

23. Rahvar AH, Haas CS, Danneberg S, Harbeck B. Increased Cardiovascular Risk in Patients with Adrenal Insufficiency: A Short Review. Biomed Res Int. 2017;2017:3691913.

24. Esposito D, Bobbio E, Di Fraia R, Mone P, Accardo G, De Bellis A, et al. Patients with adrenal insufficiency have cardiovascular features associated with hypovolemia. Endocrine. 2020;70(2):412-20.

For peer review only

For peer review only - http://bmjopen.bmj.com/site/about/guidelines.xhtml

PRISMA-P 2015 Checklist

This checklist has been adapted for use with protocol submissions to *Systematic Reviews* from Table 3 in Moher D et al: Preferred reporting items for systematic review and meta-analysis protocols (PRISMA-P) 2015 statement. *Systematic Reviews* 2015 **4**:1

Section/topic	#	Checklist item	Information reported		Line number(s)
			Yes	No	
**ADMINISTRATIVE INFORMATION**					
**Title**					
Identification	1a	Identify the report as a protocol of a systematic review	X		
Update	1b	If the protocol is for an update of a previous systematic review, identify as such		X	
**Registration**	2	If registered, provide the name of the registry (e.g., PROSPERO) and registration number in the Abstract	X		
**Authors**					
Contact	3a	Provide name, institutional affiliation, and e-mail address of all protocol authors; provide physical mailing address of corresponding author	X		
Contributions	3b	Describe contributions of protocol authors and identify the guarantor of the review	X		
**Amendments**	4	If the protocol represents an amendment of a previously completed or published protocol, identify as such and list changes; otherwise, state plan for documenting important protocol amendments		X	
**Support**					
Sources	5a	Indicate sources of financial or other support for the review	X		
Sponsor	5b	Provide name for the review funder and/or sponsor	X		
Role of sponsor/funder	5c	Describe roles of funder(s), sponsor(s), and/or institution(s), if any, in developing the protocol		X	
**INTRODUCTION**					
**Rationale**	6	Describe the rationale for the review in the context of what is already known	X		
**Objectives**	7	Provide an explicit statement of the question(s) the review will address with reference to participants, interventions, comparators, and outcomes (PICO)	X		
**METHODS**					

For peer review only

BioMed Central The Open Access Publisher

2

Section/topic	#	Checklist item	Information reported Yes	No	Line number(s)
**Eligibility criteria**	8	Specify the study characteristics (e.g., PICO, study design, setting, time frame) and report characteristics (e.g., years considered, language, publication status) to be used as criteria for eligibility for the review	X		
**Information sources**	9	Describe all intended information sources (e.g., electronic databases, contact with study authors, trial registers, or other grey literature sources) with planned dates of coverage	X		
**Search strategy**	10	Present draft of search strategy to be used for at least one electronic database, including planned limits, such that it could be repeated		X	
STUDY RECORDS					
Data management	11a	Describe the mechanism(s) that will be used to manage records and data throughout the review	X		
Selection process	11b	State the process that will be used for selecting studies (e.g., two independent reviewers) through each phase of the review (i.e., screening, eligibility, and inclusion in meta-analysis)	X		
Data collection process	11c	Describe planned method of extracting data from reports (e.g., piloting forms, done independently, in duplicate), any processes for obtaining and confirming data from investigators	X		
**Data items**	12	List and define all variables for which data will be sought (e.g., PICO items, funding sources), any pre-planned data assumptions and simplifications	X		
**Outcomes and prioritization**	13	List and define all outcomes for which data will be sought, including prioritization of main and additional outcomes, with rationale	X		
**Risk of bias in individual studies**	14	Describe anticipated methods for assessing risk of bias of individual studies, including whether this will be done at the outcome or study level, or both; state how this information will be used in data synthesis	X		
DATA					
**Synthesis**	15a	Describe criteria under which study data will be quantitatively synthesized	X		
	15b	If data are appropriate for quantitative synthesis, describe planned summary measures, methods of handling data, and methods of combining data from studies, including any planned exploration of consistency (e.g., I^2, Kendall's tau)	X		
	15c	Describe any proposed additional analyses (e.g., sensitivity or subgroup analyses, meta-regression)	X		
	15d	If quantitative synthesis is not appropriate, describe the type of summary planned		X	
**Meta-bias(es)**	16	Specify any planned assessment of meta-bias(es) (e.g., publication bias across studies, selective reporting within studies)	X		
**Confidence in cumulative evidence**	17	Describe how the strength of the body of evidence will be assessed (e.g., GRADE)	X		

BioMed Central
The Open Access Publisher

BMJ Open

Mortality in patients with adrenal insufficiency: a protocol for a systematic review and meta-analysis

Journal:	*BMJ Open*
Manuscript ID	bmjopen-2023-076582.R2
Article Type:	Protocol
Date Submitted by the Author:	19-Dec-2023
Complete List of Authors:	Allosso, Francesca; University of Campania Luigi Vanvitelli, Department of Advanced Medical and Surgical Sciences<br>Dalakas, Konstantinos; University of Gothenburg, Department of Internal Medicine and Clinical Nutrition; Sahlgrenska University Hospital, Department of Endocrinology<br>Bergthorsdottir, Ragnhildur; University of Gothenburg, Department of Internal Medicine and Clinical Nutrition; Sahlgrenska University Hospital, Department of Endocrinology<br>Chantzichristos, Dimitrios; University of Gothenburg, Department of Internal Medicine and Clinical Nutrition; Sahlgrenska University Hospital, Department of Endocrinology<br>Hessman, Eva; University of Gothenburg, Biomedical Library, Gothenburg University Library<br>Nwaru, Bright; University of Gothenburg Institute of Medicine, Krefting Research Centre; University of Gothenburg Wallenberg Centre for Molecular and Translational Medicine<br>Bobbio, Emanuele; Sahlgrenska University Hospital, Department of Cardiology; University of Gothenburg,<br>Pasquali, Daniela; University of Campania Luigi Vanvitelli, Department of Advanced Medical and Surgical Sciences<br>Johannsson, Gudmundur; University of Gothenburg, Department of Internal Medicine and Clinical Nutrition; Sahlgrenska University Hospital, Department of Endocrinology<br>Esposito, Daniela; University of Gothenburg, Department of Internal Medicine and Clinical Nutrition, Institute of Medicine, Sahlgrenska Academy; Sahlgrenska University Hospital, Department of Endocrinology
**Primary Subject Heading**:	Diabetes and endocrinology
Secondary Subject Heading:	Epidemiology
Keywords:	Pituitary disorders < DIABETES & ENDOCRINOLOGY, General endocrinology < DIABETES & ENDOCRINOLOGY, CARDIOLOGY, Adrenal disorders < DIABETES & ENDOCRINOLOGY, Mortality

SCHOLARONE™
Manuscripts

Mortality in patients with adrenal insufficiency: a protocol for a systematic review and meta-analysis

Francesca Allosso*[1], Konstantinos Dalakas*[2,3], Ragnhildur Bergthorsdottir[2,3], Dimitrios Chantzichristos[2,3], Eva Hessman[4], Bright I. Nwaru[5,6], Emanuele Bobbio[7,8], Daniela Pasquali[1], Gudmundur Johannsson[2,3], Daniela Esposito[2,3]

*These authors contributed equally

Affiliation:

[1]Department of Advanced Medical and Surgical Sciences, University of Campania "Luigi Vanvitelli", Naples, Italy; [2] Department of Internal Medicine and Clinical Nutrition, Institute of Medicine, Sahlgrenska Academy, University of Gothenburg, Gothenburg, Sweden; [3]Department of Endocrinology, Sahlgrenska University Hospital, Gothenburg, Sweden; [4]Biomedical Library, Gothenburg University Library, University of Gothenburg, Gothenburg, Sweden; [5] Krefting Research Centre, Institute of Medicine, Sahlgrenska Academy, University of Gothenburg, Gothenburg, Sweden; [6] Wallenberg Centre for Molecular and Translational Medicine, Institute of Medicine, University of Gothenburg, Gothenburg, Sweden; [7]Department of Cardiology, Sahlgrenska University Hospital, Gothenburg, Sweden; [8]Institute of Medicine at Sahlgrenska Academy, University of Gothenburg, Gothenburg, Sweden

Key Words: adrenal insufficiency, hypopituitarism, mortality, glucocorticoid replacement

Abstract: 232 words

Manuscript: 2273 words

References: 24

Running title: Mortality in adrenal insufficiency

For peer review only

Corresponding author and person to whom reprint requests should be addressed:

Daniela Esposito, MD, PhD, Department of Endocrinology, Sahlgrenska University Hospital Institute of Medicine, Sahlgrenska Academy, University of Gothenburg, Gothenburg, Sweden. Email: daniela.esposito@gu.se

For peer review only

For peer review only - http://bmjopen.bmj.com/site/about/guidelines.xhtml

Abstract

Introduction

Adrenal insufficiency (AI) is a rare disorder characterized by an impaired secretion of glucocorticoids from the adrenal glands. Treatment strategies for AI have developed over time with reduced glucocorticoid replacement doses and improved circadian exposure regimens, but whether this has resulted in better survival is unknown.

The main purpose of this systematic review is to gather and synthesize available evidence on long-term mortality in patients with AI. Secondary aim is to study causes of death, with focus on cardiovascular and infectious diseases, in AI patients.

Methods and analysis

Studies published from the inception of respective databases (Medline, Embase, Cochrane and Web of Science) until the end of May 2023 will be systematically synthetized. Observational studies with a reference population will be included, and their quality will be assessed using the Newcastle-Ottawa scale. Data collected will be narratively integrated and a meta-analysis will be performed to pool data from studies considered homogenous. The systematic review will be reported following the PRISMA guidelines. This will be the first systematic review assessing mortality and causes of death in AI patients. The findings of this systematic review will be of value for both patients and healthcare providers.

Ethics and dissemination

This systematic review does not require ethical approval or informed consent because it will be based on previously published data only and does not implicate any direct contact with individual patients.

PROSPERO registration number: CRD42023416253

For peer review only

Strengths and limitations of this study

- This systematic review will for the first time provide a comprehensive synthesis of overall mortality in patients with adrenal insufficiency.
- The protocol of this systematic review follows the Preferred Reporting Items for Systematic Review and Meta-Analysis Protocols guidelines.
- A detailed description of the review processes is presented in this protocol increasing the reproducibility of the systematic review.
- All available studies on mortality in adrenal insufficiency will be gathered from publicly available literature databases, without language or geographical limitations.
- A potential limitation can be high heterogeneity in the population, as both primary and secondary adrenal insufficiency are going to be included. However, separate sub-group analyses will be performed.

For peer review only

For peer review only - http://bmjopen.bmj.com/site/about/guidelines.xhtml

Introduction

Adrenal insufficiency (AI) is a rare disorder characterized by an impaired secretion of glucocorticoids from the adrenal cortex (1). Primary AI (PAI), with an estimated prevalence of 93-144/million in Europe, is caused by disorders of the adrenal glands and is usually associated with mineralocorticoid and androgen deficiency (1). It can be acquired or inherited as in congenital adrenal hyperplasia (CAH), usually due to steroid 21-hydroxylase deficiency (2). In western countries acquired PAI is most commonly due to an autoimmune-mediated adrenalitis, which can be isolated or combined with other autoimmune diseases (e.g. autoimmune polyglandular syndrome type 1 or 2) (2, 3). Other causes of PAI are tuberculosis and infectious diseases (3). Secondary AI (SAI), with an estimated prevalence of 150-280/million, is caused by adrenocorticotropic hormone (ACTH) deficiency secondary to a pituitary disease and is often associated with other pituitary hormone deficiencies (1, 2).

Treatment of AI consists of replacement therapy with glucocorticoids (GC) (1). In PAI, mineralocorticoid replacement therapy is also required while in SAI additional replacement therapy with thyroid hormone, sex steroids and growth hormone may be needed due to failure of other pituitary hormone axes (2). Before the introduction of GC therapy, most patients with PAI died within 2 years of diagnosis (4). Although long-term outcomes have markedly improved, mortality remains increased, with cardiovascular disease being the leading cause of death (5-7).

Standard replacement treatments in AI include short-acting glucocorticoids such as hydrocortisone and cortisone acetate, usually given 2-3 times daily (8). In some countries other GCs such as prednisolone are used (2). However, conventional GC replacement fails to restore a circadian cortisol profile in patients with AI.

There have been attempts to improve the dosing regimen of GC replacement therapy and the management of intercurrent illnesses (9). The introduction of new therapies, as once-daily modified release hydrocortisone that better mimics the endogen circadian production of cortisol, has been associated with a more favourable metabolic outcome (10). Whether this has led to improvement in survival rate is however unknown. Data on mortality in patients with AI are discordant (6, 11-14). Despite having GC deficiency in common, PAI and SAI have

For peer review only - http://bmjopen.bmj.com/site/about/guidelines.xhtml

different aetiologies. SAI is usually associated with other pituitary deficiencies such as growth hormone deficiency, which per se could lead to increased risk of cardiovascular diseases and death (15). On the other hand, PAI is often associated with other autoimmune diseases which may play an important role on patients' outcome (16).

No previous studies have so far synthesized the available evidence on mortality in patients with AI. In this systematic review, we will analyse overall and disease specific mortality in patients with PAI and SAI.

Study aims and objectives

The primary aim of this systematic review is to identify, critically appraise and synthetize available data on long-term mortality in adult patients with AI. Secondary aims include the assessment of mortality due to cardiovascular and infectious diseases.

Methods and analysis

The protocol for this review follows the recommendation of the preferred reporting items for systematic review and meta-analysis protocols (PRISMA-P) statement and has been registered in PROSPERO with registration number: CRD42023416253 (17).

This systematic review was planned in November 2022 and is expected to be finalized in June 2024. In the final systematic review, we will describe any deviation from the protocol. The final systematic review will be reported according to the PRISMA guidelines (17).

Eligibility criteria

The population of interest will be adult patients with AI, and this includes patients with PAI (including CAH) and SAI. Studies reporting long-term mortality (defined as death occurring ≥ 1 year from the diagnosis of AI) will be eligible for inclusion. Data across different age groups, gender, and ethnicity will be analyzed, and there will be no exclusion by country or language. Patients with Waterhouse-Friderichsen syndrome, leukodystrophy and Type 4 Renal tubular acidosis will be excluded from the analysis. Studies not including a comparison group (i.e. a background population) will be excluded. In addition, review articles, non-peer reviewed

For peer review only - http://bmjopen.bmj.com/site/about/guidelines.xhtml

For peer review only

articles, commentaries, proceedings, laboratory science studies, case studies, case series, and other studies that do not allow the calculation of rates of the outcomes will be excluded from this systematic review. If an eligible study does not report the incidence of the prespecified outcomes, we will attempt to contact the study authors to request for the respective details.

Information sources

Our data sources will include electronic databases, conference abstracts, gray literature, and researchers and authors themselves. A systematic search will be performed through Medline, Embase, Cochrane and Web of Science using commonly used phrases stated in related literature, in addition to controlled vocabulary, i.e. Medical Subject Headings (MeSH) terms in Ovid Medline and Cochrane Central, and Emtree terms in Embase. The databases will be searched from their inception date until the end of May 2023. Broad and inclusive search terms will be used in order to limit the risk of omitting any relevant studies.

We will review reference lists and citations of included studies in order to find additional relevant articles. In addition, we will perform an updated search at the completion of the systematic review to ensure that studies published after the first search will be included.

Search strategy

Our initial search syntax for Ovid Medline is given below and this will be adapted in searching the other databases.

1 Adrenal Insufficiency/ or Addison Disease/ or Hypoadrenocorticism, Familial/ or Adrenoleukodystrophy/ or Hypoaldosteronism/ or Waterhouse-Friedrichsen syndrome/ or Hypopituitarism/ or Adrenal Hyperplasia, Congenital/

2 ('Adrenal Insufficiency' or Hypoadrenalism or 'Adrenal Gland Hypofunction' or Addison* or 'Adrenocortical Insufficiency' or 'Familial Hypoadrenocorticism' or Adrenoleukodystrophy or Hypoaldosteronism or 'Waterhouse-Friedrichsen syndrome' or 'Complex Glycerol Kinase Deficiency' or 'Xp21 Contiguous Gene Deletion Syndrome' or 'X-

For peer review only - http://bmjopen.bmj.com/site/about/guidelines.xhtml

linked Adrenal Hypoplasia' or 'X-Linked Addison Disease' or 'Cytomegalic Adrenocortical Hypoplasia' or 'Congenital Adrenal Hypoplasia' or 'Type IV Renal Tubular Acidosis' or 'Purpura Fulminans' or 'Meningococcal Hemorrhagic Adrenalitis' or Hypopituitarism or 'Congenital Adrenal Hyperplasia').tw,kf.

3 (mortality or death* or ((fatal or long-term) adj2 outcome*)).tw,kf. /freq=2

4 Mortality/ or Death/ or Survival/ or Fatal Outcome/ or Mortality.fs.

5 (exp animal/ or exp invertebrate/ or animal experiment/ or animal model/ or exp plant/ or exp fungus/) not exp human/

6 1 or 2

7 3 or 4

8 6 and 7

9 8 not 5

Selection process

Articles will be independently screened by two reviewers using the Rayyan web application for systematic reviews. First, articles will be screened by titles and/or abstracts. The screened articles will be classified into three groups: relevant, irrelevant, and uncertain. Articles that both reviewers consider to be irrelevant will be excluded from the review. For articles that are considered relevant or uncertain based on their title and/or abstract, the full text article will be retrieved to assess eligibility. Potentially relevant articles selected by at least one author will be investigated and evaluated in full. Next, the full text of the eligible articles will be reviewed and a list of articles to be included by each reviewer will be created. The two lists will then be compared, and the discrepancy will be discussed. If no agreement is reached, the entire team will make the final decision. A flow diagram showing the selection process will be created using the PRISMA guidelines.

For peer review only

For peer review only - http://bmjopen.bmj.com/site/about/guidelines.xhtml

Data extraction

Predefined information of interest from included studies will be extracted using a data extraction form. This will help standardize the information recorded and aid analyses. The following data will be extracted independently by two reviewers: study name (along with the name of the first author and year of publication), country where the study was conducted, study size, source from which patients or study participants were selected, study design, definition of AI, definition of CAH, disease duration, outcomes definition, age, gender, ethnicity, incidence or prevalence with 95% confidence intervals (CIs) or the number of patients achieving the outcomes. Data on causes of PAI and SAI will be systematically gathered, as underlying conditions themselves may independently influence the outcomes. Data on doses of CG and MC will be collected, as treatment strategies have changed over time. Studies including patients with hypopituitarism will be included with the aim to gather data on patients with SAI. If the required data are inadequate, unclear, or missing from the article, authors will be contacted. If needed, missing information will be calculated from the available data when possible. In case of studies with overlapping population, only the study with the largest sample size and higher quality will be included. The data extraction will be discussed by two reviewers, and the entire team will arbitrate in the case of any disagreement.

Quality assessment

The quality of the studies will be independently assessed by two authors using the Newcastle–Ottawa quality assessment scale (NOS). Critical appraisal of articles will be performed to assess the methodological quality of each article. The quality appraisal will be undertaken by two reviewers and a third reviewer will arbitrate in the case of any disagreement.

Data synthesis

Characteristics and main results of the included studies will be presented in descriptive tables and the gathered evidence will be synthesized narratively.

For peer review only - http://bmjopen.bmj.com/site/about/guidelines.xhtml

We will calculate rate of mortality in each study if possible. If the included studies are sufficiently homogeneous, we will undertake a meta-analysis by pooling estimates across studies using the random effects approach.

Heterogeneity between studies will be assessed using the Higgins I^2 statistic (18). I^2 values of 0–30% will be considered as minimal heterogeneity, 31–50% moderate heterogeneity, and > 50% substantial heterogeneity (18). As PAI and SAI are two different entities, we will conduct separate subanalyses on mortality in PAI and SAI. If data are sufficient, we will conduct subgroup analysis to explore potential reasons for heterogeneity based on the quality of study, country, age, gender, and ethnicity. A subgroup analysis will be also conduct for patients with CAH.

Begg's funnel plot and Egger's test will be used to assess publication bias (19). The former is a scatter plot of the log odds ratios (ORs) of individual studies on the x-axis against 1/ standard error (SE) of each study on the y-axis(19). Egger's test is a linear regression test of the normalized effect estimate (log OR/SE) against its precision (1/SE) (19). P value of < 0.10 on Egger's test or an asymmetrical funnel plot will be considered to indicate the presence of publication bias.

Ethical issues

This systematic review does not require ethical approval or informed consent because it will be based only on previously published data and does not implicate any direct contact with individual patients.

Patient and public involvement

No patient will be involved in this systematic review

Discussion

Adrenal insufficiency is a life-threatening disorder. In the past, most patients with AI died within a few years after diagnosis due to scarce treatment options (3). Thanks to the discovery

For peer review only - http://bmjopen.bmj.com/site/about/guidelines.xhtml

of cortisone in 1940s, the survival of patients with AI has improved (3). In the 1960s, Mason et al. showed that PAI patients on GC replacement therapy had a mortality rate similar to that of the background population (20). In line with that study, a recent Norwegian study, including 811 patients with PAI, showed no difference in mortality rate between PAI patients and the general Norwegian population (11). Conversely, our group showed that mortality in PAI patients was 2-fold increased in comparison with the general Swedish population, after following 1675 patients with PAI between 1987 and 2001 (6). More recent data from UK have confirmed this finding showing a more than 2-fold excess mortality in patients with PAI (16).

Mortality in AI patients has been related to cardiovascular diseases, infections, acute adrenal crises, and malignancies (7). In a large retrospective cohort study including 6821 patients with AI from United Kingdom, Ngaosuwan et al. have recently shown that overall mortality is higher by approximately 80% in PAI and 50% in SAI in comparison with matched controls (7). Another study on the same cohort has showed that mortality due to cardiovascular disease was increased in both PAI and SAI compared with matched controls (21). Interestingly, patients with SAI had an increased risk of cerebrovascular events when compared with PAI patients (21).

Other common causes of death in AI are infectious diseases (7). A multicenter study including 2034 patients with PAI and SAI from the European Adrenal Insufficiency Registry (EU-AIR) showed that the major causes of death were cardiovascular disease (35%) and infectious diseases (15%)(22). In a population-based study, including 3299 patients with PAI, Bensing *et al.* have found that the risk of death due to infections was 6-fold increased compared to the general population (5). Moreover, in a cohort study of 1286 hypopituitary patients, Burman *et al.* have found that mortality from infections was increased in ACTH-deficient patients but not in patients without ACTH deficiency (12).

Inappropriate replacement therapy with GCs has been described as the main determinant of a poor prognosis in AI patients (23). Excessive cortisol exposure and glucocorticoid replacement therapy that does not mimic cortisol circadian rhythm are indeed associated with the adverse metabolic profile, increased morbidity, and mortality (23, 24).

Over the last two decades, efforts have been made to optimize GC treatment, trying to reduce the total daily GC dose and optimise intercurrent illness management (9). In addition, novel treatment strategies have been introduced to better mimic the physiological diurnal variation in cortisol levels (10). Whether this has led to an improvement in long-term outcomes remains

For peer review only - http://bmjopen.bmj.com/site/about/guidelines.xhtml

unknown. Previous studies conducted on this topic have shown controversial findings due to different study designs, populations and settings (5, 6, 11). Since AI is a rare disease, no single center has accumulated sufficient experience to draw definitive conclusions. Therefore, a systematic review is needed to provide clearer insights into this topic. Increasing knowledge of AI mortality can lead to optimized management of AI and its comorbidities.

Contributions

DE conceived this study. FA, KD, DP and DE developed the study protocol. All the authors revised the study protocol. FA and KD will implement the systematic review under the supervision of DE, EB, RB, DC, BN, DP and GJ. EH will develop the search strings and conduct the study search. EB and BN will provide the statistical analysis expertise. EB, BN, and DE will conduct data analysis. FA and KD will perform the screening and extraction of data whereas DE, EB, RB, DC, BN and GJ will review the work.

Funding statement

This work received support from the Swedish state under the agreement between the Swedish government and the county councils, the ALF-agreement (ALFGBG-874631 and ALFGBG-960884).

Conflicts of interest and financial disclosure

None declared

For peer review only - http://bmjopen.bmj.com/site/about/guidelines.xhtml

References

1. Charmandari E, Nicolaides NC, Chrousos GP. Adrenal insufficiency. Lancet. 2014;383(9935):2152-67.
2. Husebye ES, Pearce SH, Krone NP, Kampe O. Adrenal insufficiency. Lancet. 2021;397(10274):613-29.
3. Kumar R, Wassif WS. Adrenal insufficiency. J Clin Pathol. 2022;75(7):435-42.
4. Dunlop D. Eighty-Six Cases of Addison's Disease. Br Med J. 1963;2(5362):887-91.
5. Bensing S, Brandt L, Tabaroj F, Sjoberg O, Nilsson B, Ekbom A, et al. Increased death risk and altered cancer incidence pattern in patients with isolated or combined autoimmune primary adrenocortical insufficiency. Clin Endocrinol (Oxf). 2008;69(5):697-704.
6. Bergthorsdottir R, Leonsson-Zachrisson M, Oden A, Johannsson G. Premature mortality in patients with Addison's disease: a population-based study. J Clin Endocrinol Metab. 2006;91(12):4849-53.
7. Ngaosuwan K, Johnston DG, Godsland IF, Cox J, Majeed A, Quint JK, et al. Increased Mortality Risk in Patients With Primary and Secondary Adrenal Insufficiency. J Clin Endocrinol Metab. 2021;106(7):e2759-e68.
8. Ngaosuwan K, Johnston DG, Godsland IF, Cox J, Majeed A, Quint JK, et al. Mortality Risk in Patients With Adrenal Insufficiency Using Prednisolone or Hydrocortisone: A Retrospective Cohort Study. J Clin Endocrinol Metab. 2021;106(8):2242-51.
9. Johannsson G, Skrtic S, Lennernas H, Quinkler M, Stewart PM. Improving outcomes in patients with adrenal insufficiency: a review of current and future treatments. Curr Med Res Opin. 2014;30(9):1833-47.
10. Johannsson G, Nilsson AG, Bergthorsdottir R, Burman P, Dahlqvist P, Ekman B, et al. Improved cortisol exposure-time profile and outcome in patients with adrenal insufficiency: a prospective randomized trial of a novel hydrocortisone dual-release formulation. J Clin Endocrinol Metab. 2012;97(2):473-81.
11. Erichsen MM, Lovas K, Fougner KJ, Svartberg J, Hauge ER, Bollerslev J, et al. Normal overall mortality rate in Addison's disease, but young patients are at risk of premature death. Eur J Endocrinol. 2009;160(2):233-7.
12. Burman P, Mattsson AF, Johannsson G, Hoybye C, Holmer H, Dahlqvist P, et al. Deaths among adult patients with hypopituitarism: hypocortisolism during acute stress, and de novo malignant brain tumors contribute to an increased mortality. J Clin Endocrinol Metab. 2013;98(4):1466-75.
13. O'Reilly MW, Reulen RC, Gupta S, Thompson CA, Dineen R, Goulden EL, et al. ACTH and gonadotropin deficiencies predict mortality in patients treated for nonfunctioning pituitary adenoma: long-term follow-up of 519 patients in two large European centres. Clin Endocrinol (Oxf). 2016;85(5):748-56.
14. Hammarstrand C, Ragnarsson O, Hallen T, Andersson E, Skoglund T, Nilsson AG, et al. Higher glucocorticoid replacement doses are associated with increased mortality in patients with pituitary adenoma. Eur J Endocrinol. 2017;177(3):251-6.
15. Gola M, Bonadonna S, Doga M, Giustina A. Clinical review: Growth hormone and cardiovascular risk factors. J Clin Endocrinol Metab. 2005;90(3):1864-70.
16. Conrad N, Verbeke G, Molenberghs G, Goetschalckx L, Callender T, Cambridge G, et al. Autoimmune diseases and cardiovascular risk: a population-based study on 19 autoimmune diseases and 12 cardiovascular diseases in 22 million individuals in the UK. Lancet. 2022;400(10354):733-43.

For peer review only - http://bmjopen.bmj.com/site/about/guidelines.xhtml

17. Shamseer L, Moher D, Clarke M, Ghersi D, Liberati A, Petticrew M, et al. Preferred reporting items for systematic review and meta-analysis protocols (PRISMA-P) 2015: elaboration and explanation. BMJ. 2015;350:g7647.
18. Higgins JP, Thompson SG. Quantifying heterogeneity in a meta-analysis. Stat Med. 2002;21(11):1539-58.
19. Egger M, Smith GD. Bias in location and selection of studies. BMJ. 1998;316(7124):61-6.
20. Mason AS, Meade TW, Lee JA, Morris JN. Epidemiological and clinical picture of Addison's disease. Lancet. 1968;2(7571):744-7.
21. Ngaosuwan K, Johnston DG, Godsland IF, Cox J, Majeed A, Quint JK, et al. Cardiovascular Disease in Patients With Primary and Secondary Adrenal Insufficiency and the Role of Comorbidities. J Clin Endocrinol Metab. 2021;106(5):1284-93.
22. Quinkler M, Ekman B, Zhang P, Isidori AM, Murray RD, Investigators E-A. Mortality data from the European Adrenal Insufficiency Registry-Patient characterization and associations. Clin Endocrinol (Oxf). 2018;89(1):30-5.
23. Rahvar AH, Haas CS, Danneberg S, Harbeck B. Increased Cardiovascular Risk in Patients with Adrenal Insufficiency: A Short Review. Biomed Res Int. 2017;2017:3691913.
24. Esposito D, Bobbio E, Di Fraia R, Mone P, Accardo G, De Bellis A, et al. Patients with adrenal insufficiency have cardiovascular features associated with hypovolemia. Endocrine. 2020;70(2):412-20.

For peer review only

For peer review only - http://bmjopen.bmj.com/site/about/guidelines.xhtml

1

PRISMA-P 2015 Checklist

This checklist has been adapted for use with protocol submissions to *Systematic Reviews* from Table 3 in Moher D et al: Preferred reporting items for systematic review and meta-analysis protocols (PRISMA-P) 2015 statement. *Systematic Reviews* 2015 **4**:1

Section/topic	#	Checklist item	Information reported		Line number(s)
			Yes	No	
**ADMINISTRATIVE INFORMATION**					
**Title**					
Identification	1a	Identify the report as a protocol of a systematic review	X		
Update	1b	If the protocol is for an update of a previous systematic review, identify as such		X	
**Registration**	2	If registered, provide the name of the registry (e.g., PROSPERO) and registration number in the Abstract	X		
**Authors**					
Contact	3a	Provide name, institutional affiliation, and e-mail address of all protocol authors; provide physical mailing address of corresponding author	X		
Contributions	3b	Describe contributions of protocol authors and identify the guarantor of the review	X		
**Amendments**	4	If the protocol represents an amendment of a previously completed or published protocol, identify as such and list changes; otherwise, state plan for documenting important protocol amendments		X	
**Support**					
Sources	5a	Indicate sources of financial or other support for the review	X		
Sponsor	5b	Provide name for the review funder and/or sponsor	X		
Role of sponsor/funder	5c	Describe roles of funder(s), sponsor(s), and/or institution(s), if any, in developing the protocol		X	
**INTRODUCTION**					
**Rationale**	6	Describe the rationale for the review in the context of what is already known	X		
**Objectives**	7	Provide an explicit statement of the question(s) the review will address with reference to participants, interventions, comparators, and outcomes (PICO)	X		
**METHODS**					

BioMed Central The Open Access Publisher

Section/topic	#	Checklist item	Information reported		Line number(s)
			Yes	No	
**Eligibility criteria**	8	Specify the study characteristics (e.g., PICO, study design, setting, time frame) and report characteristics (e.g., years considered, language, publication status) to be used as criteria for eligibility for the review	X		
**Information sources**	9	Describe all intended information sources (e.g., electronic databases, contact with study authors, trial registers, or other grey literature sources) with planned dates of coverage	X		
**Search strategy**	10	Present draft of search strategy to be used for at least one electronic database, including planned limits, such that it could be repeated		X	
STUDY RECORDS					
Data management	11a	Describe the mechanism(s) that will be used to manage records and data throughout the review	X		
Selection process	11b	State the process that will be used for selecting studies (e.g., two independent reviewers) through each phase of the review (i.e., screening, eligibility, and inclusion in meta-analysis)	X		
Data collection process	11c	Describe planned method of extracting data from reports (e.g., piloting forms, done independently, in duplicate), any processes for obtaining and confirming data from investigators	X		
**Data items**	12	List and define all variables for which data will be sought (e.g., PICO items, funding sources), any pre-planned data assumptions and simplifications	X		
**Outcomes and prioritization**	13	List and define all outcomes for which data will be sought, including prioritization of main and additional outcomes, with rationale	X		
**Risk of bias in individual studies**	14	Describe anticipated methods for assessing risk of bias of individual studies, including whether this will be done at the outcome or study level, or both; state how this information will be used in data synthesis	X		
DATA					
**Synthesis**	15a	Describe criteria under which study data will be quantitatively synthesized	X		
	15b	If data are appropriate for quantitative synthesis, describe planned summary measures, methods of handling data, and methods of combining data from studies, including any planned exploration of consistency (e.g., I^2, Kendall's tau)	X		
	15c	Describe any proposed additional analyses (e.g., sensitivity or subgroup analyses, meta-regression)	X		
	15d	If quantitative synthesis is not appropriate, describe the type of summary planned		X	
**Meta-bias(es)**	16	Specify any planned assessment of meta-bias(es) (e.g., publication bias across studies, selective reporting within studies)	X		
**Confidence in cumulative evidence**	17	Describe how the strength of the body of evidence will be assessed (e.g., GRADE)	X		

For peer review only

BioMed Central The Open Access Publisher

BMJ Open

Mortality in patients with adrenal insufficiency: a protocol for a systematic review and meta-analysis

Journal:	*BMJ Open*
Manuscript ID	bmjopen-2023-076582.R3
Article Type:	Protocol
Date Submitted by the Author:	12-Jan-2024
Complete List of Authors:	Allosso, Francesca; University of Campania Luigi Vanvitelli, Department of Advanced Medical and Surgical Sciences<br>Dalakas, Konstantinos; University of Gothenburg, Department of Internal Medicine and Clinical Nutrition; Sahlgrenska University Hospital, Department of Endocrinology<br>Bergthorsdottir, Ragnhildur; University of Gothenburg, Department of Internal Medicine and Clinical Nutrition; Sahlgrenska University Hospital, Department of Endocrinology<br>Chantzichristos, Dimitrios; University of Gothenburg, Department of Internal Medicine and Clinical Nutrition; Sahlgrenska University Hospital, Department of Endocrinology<br>Hessman, Eva; University of Gothenburg, Biomedical Library, Gothenburg University Library<br>Nwaru, Bright; University of Gothenburg Institute of Medicine, Krefting Research Centre; University of Gothenburg Wallenberg Centre for Molecular and Translational Medicine<br>Bobbio, Emanuele; Sahlgrenska University Hospital, Department of Cardiology; University of Gothenburg,<br>Pasquali, Daniela; University of Campania Luigi Vanvitelli, Department of Advanced Medical and Surgical Sciences<br>Johannsson, Gudmundur; University of Gothenburg, Department of Internal Medicine and Clinical Nutrition; Sahlgrenska University Hospital, Department of Endocrinology<br>Esposito, Daniela; University of Gothenburg, Department of Internal Medicine and Clinical Nutrition, Institute of Medicine, Sahlgrenska Academy; Sahlgrenska University Hospital, Department of Endocrinology
**Primary Subject Heading**:	Diabetes and endocrinology
Secondary Subject Heading:	Epidemiology
Keywords:	Pituitary disorders < DIABETES & ENDOCRINOLOGY, General endocrinology < DIABETES & ENDOCRINOLOGY, CARDIOLOGY, Adrenal disorders < DIABETES & ENDOCRINOLOGY, Mortality

SCHOLARONE™
Manuscripts

Mortality in patients with adrenal insufficiency: a protocol for a systematic review and meta-analysis

Francesca Allosso*[1], Konstantinos Dalakas*[2,3], Ragnhildur Bergthorsdottir[2,3], Dimitrios Chantzichristos[2,3], Eva Hessman[4], Bright I. Nwaru[5,6], Emanuele Bobbio[7,8], Daniela Pasquali[1], Gudmundur Johannsson[2,3], Daniela Esposito[2,3]

*These authors contributed equally

Affiliation:

[1]Department of Advanced Medical and Surgical Sciences, University of Campania “Luigi Vanvitelli”, Naples, Italy; [2] Department of Internal Medicine and Clinical Nutrition, Institute of Medicine, Sahlgrenska Academy, University of Gothenburg, Gothenburg, Sweden; [3]Department of Endocrinology, Sahlgrenska University Hospital, Gothenburg, Sweden; [4]Biomedical Library, Gothenburg University Library, University of Gothenburg, Gothenburg, Sweden; [5] Krefting Research Centre, Institute of Medicine, Sahlgrenska Academy, University of Gothenburg, Gothenburg, Sweden; [6] Wallenberg Centre for Molecular and Translational Medicine, Institute of Medicine, University of Gothenburg, Gothenburg, Sweden; [7]Department of Cardiology, Sahlgrenska University Hospital, Gothenburg, Sweden; [8]Institute of Medicine at Sahlgrenska Academy, University of Gothenburg, Gothenburg, Sweden

Key Words: adrenal insufficiency, hypopituitarism, mortality, glucocorticoid replacement

Abstract: 248 words

Manuscript: 2294 words

References: 24

Running title: Mortality in adrenal insufficiency

For peer review only

For peer review only - http://bmjopen.bmj.com/site/about/guidelines.xhtml

Corresponding author and person to whom reprint requests should be addressed:
Daniela Esposito, MD, PhD, Department of Endocrinology, Sahlgrenska University Hospital Institute of Medicine, Sahlgrenska Academy, University of Gothenburg, Gothenburg, Sweden. Email: daniela.esposito@gu.se

For peer review only

Abstract

Introduction

Adrenal insufficiency (AI) is a rare disorder characterized by an impaired secretion of glucocorticoids from the adrenal glands. Treatment strategies for AI have developed over time with reduced glucocorticoid replacement doses and improved circadian exposure regimens, but whether this has resulted in better survival is unknown.

The main purpose of this systematic review is to gather and synthesize available evidence on long-term mortality in patients with AI. Secondary aim is to study causes of death, with focus on cardiovascular and infectious diseases, in AI patients.

Methods and analysis

Studies published from the inception of respective databases (Medline, Embase, Cochrane and Web of Science) until the end of May 2023 will be systematically synthetized. Observational studies with a reference population will be included, and their quality will be assessed using the Newcastle-Ottawa scale. Data collected will be narratively integrated and a meta-analysis will be performed to pool data from studies considered homogenous. The systematic review will be reported following the PRISMA guidelines. This will be the first systematic review assessing mortality and causes of death in AI patients. The findings of this systematic review will be of value for both patients and healthcare providers.

Ethics and dissemination

This systematic review does not require ethical approval or informed consent because it will be based on previously published data only and does not implicate any direct contact with individual patients. The research results will be presented at scientific conferences and submitted for publication in an internationally recognized peer-reviewed scientific journal.

PROSPERO registration number: CRD42023416253

For peer review only

Strengths and limitations of this study

- This systematic review will for the first time provide a comprehensive synthesis of overall mortality in patients with adrenal insufficiency.
- The protocol of this systematic review follows the Preferred Reporting Items for Systematic Review and Meta-Analysis Protocols guidelines.
- A detailed description of the review processes is presented in this protocol increasing the reproducibility of the systematic review.
- All available studies on mortality in adrenal insufficiency will be gathered from publicly available literature databases, without language or geographical limitations.
- A potential limitation can be high heterogeneity in the population, as both primary and secondary adrenal insufficiency are going to be included. However, separate sub-group analyses will be performed.

For peer review only

Introduction

Adrenal insufficiency (AI) is a rare disorder characterized by an impaired secretion of glucocorticoids from the adrenal cortex (1). Primary AI (PAI), with an estimated prevalence of 93-144/million in Europe, is caused by disorders of the adrenal glands and is usually associated with mineralocorticoid and androgen deficiency (1). It can be acquired or inherited as in congenital adrenal hyperplasia (CAH), usually due to steroid 21-hydroxylase deficiency (2). In western countries acquired PAI is most commonly due to an autoimmune-mediated adrenalitis, which can be isolated or combined with other autoimmune diseases (e.g. autoimmune polyglandular syndrome type 1 or 2) (2, 3). Other causes of PAI are tuberculosis and infectious diseases (3). Secondary AI (SAI), with an estimated prevalence of 150-280/million, is caused by adrenocorticotropic hormone (ACTH) deficiency secondary to a pituitary disease and is often associated with other pituitary hormone deficiencies (1, 2).

Treatment of AI consists of replacement therapy with glucocorticoids (GC) (1). In PAI, mineralocorticoid replacement therapy is also required while in SAI additional replacement therapy with thyroid hormone, sex steroids and growth hormone may be needed due to failure of other pituitary hormone axes (2). Before the introduction of GC therapy, most patients with PAI died within 2 years of diagnosis (4). Although long-term outcomes have markedly improved, mortality remains increased, with cardiovascular disease being the leading cause of death (5-7).

Standard replacement treatments in AI include short-acting glucocorticoids such as hydrocortisone and cortisone acetate, usually given 2-3 times daily (8). In some countries other GCs such as prednisolone are used (2). However, conventional GC replacement fails to restore a circadian cortisol profile in patients with AI.

There have been attempts to improve the dosing regimen of GC replacement therapy and the management of intercurrent illnesses (9). The introduction of new therapies, as once-daily modified release hydrocortisone that better mimics the endogen circadian production of cortisol, has been associated with a more favourable metabolic outcome (10). Whether this has led to improvement in survival rate is however unknown. Data on mortality in patients with AI are discordant (6, 11-14). Despite having GC deficiency in common, PAI and SAI have

For peer review only - http://bmjopen.bmj.com/site/about/guidelines.xhtml

different aetiologies. SAI is usually associated with other pituitary deficiencies such as growth hormone deficiency, which per se could lead to increased risk of cardiovascular diseases and death (15). On the other hand, PAI is often associated with other autoimmune diseases which may play an important role on patients' outcome (16).

No previous studies have so far synthesized the available evidence on mortality in patients with AI. In this systematic review, we will analyse overall and disease specific mortality in patients with PAI and SAI.

Study aims and objectives

The primary aim of this systematic review is to identify, critically appraise and synthetize available data on long-term mortality in adult patients with AI. Secondary aims include the assessment of mortality due to cardiovascular and infectious diseases.

Methods and analysis

The protocol for this review follows the recommendation of the preferred reporting items for systematic review and meta-analysis protocols (PRISMA-P) statement and has been registered in PROSPERO with registration number: CRD42023416253 (17).

This systematic review was planned in November 2022 and is expected to be finalized in June 2024. In the final systematic review, we will describe any deviation from the protocol.

The final systematic review will be reported according to the PRISMA guidelines (17).

Eligibility criteria

The population of interest will be adult patients with AI, and this includes patients with PAI (including CAH) and SAI. Studies reporting long-term mortality (defined as death occurring ≥ 1 year from the diagnosis of AI) will be eligible for inclusion. Data across different age groups, gender, and ethnicity will be analyzed, and there will be no exclusion by country or language. Patients with Waterhouse-Friderichsen syndrome, leukodystrophy and Type 4 Renal tubular acidosis will be excluded from the analysis. Studies not including a comparison group (i.e. a background population) will be excluded. In addition, review articles, non-peer reviewed

For peer review only

For peer review only - http://bmjopen.bmj.com/site/about/guidelines.xhtml

articles, commentaries, proceedings, laboratory science studies, case studies, case series, and other studies that do not allow the calculation of rates of the outcomes will be excluded from this systematic review. If an eligible study does not report the incidence of the prespecified outcomes, we will attempt to contact the study authors to request for the respective details.

Information sources

Our data sources will include electronic databases, conference abstracts, gray literature, and researchers and authors themselves. A systematic search will be performed through Medline, Embase, Cochrane and Web of Science using commonly used phrases stated in related literature, in addition to controlled vocabulary, i.e. Medical Subject Headings (MeSH) terms in Ovid Medline and Cochrane Central, and Emtree terms in Embase. The databases will be searched from their inception date until the end of May 2023. Broad and inclusive search terms will be used in order to limit the risk of omitting any relevant studies.

We will review reference lists and citations of included studies in order to find additional relevant articles. In addition, we will perform an updated search at the completion of the systematic review to ensure that studies published after the first search will be included.

Search strategy

Our initial search syntax for Ovid Medline is given below and this will be adapted in searching the other databases.

1 Adrenal Insufficiency/ or Addison Disease/ or Hypoadrenocorticism, Familial/ or Adrenoleukodystrophy/ or Hypoaldosteronism/ or Waterhouse-Friedrichsen syndrome/ or Hypopituitarism/ or Adrenal Hyperplasia, Congenital/

2 ('Adrenal Insufficiency' or Hypoadrenalism or 'Adrenal Gland Hypofunction' or Addison* or 'Adrenocortical Insufficiency' or 'Familial Hypoadrenocorticism' or Adrenoleukodystrophy or Hypoaldosteronism or 'Waterhouse-Friedrichsen syndrome' or 'Complex Glycerol Kinase Deficiency' or 'Xp21 Contiguous Gene Deletion Syndrome' or 'X-

For peer review only - http://bmjopen.bmj.com/site/about/guidelines.xhtml

linked Adrenal Hypoplasia' or 'X-Linked Addison Disease' or 'Cytomegalic Adrenocortical Hypoplasia' or 'Congenital Adrenal Hypoplasia' or 'Type IV Renal Tubular Acidosis' or 'Purpura Fulminans' or 'Meningococcal Hemorrhagic Adrenalitis' or Hypopituitarism or 'Congenital Adrenal Hyperplasia').tw,kf.

3 (mortality or death* or ((fatal or long-term) adj2 outcome*)).tw,kf. /freq=2

4 Mortality/ or Death/ or Survival/ or Fatal Outcome/ or Mortality.fs.

5 (exp animal/ or exp invertebrate/ or animal experiment/ or animal model/ or exp plant/ or exp fungus/) not exp human/

6 1 or 2

7 3 or 4

8 6 and 7

9 8 not 5

Selection process

Articles will be independently screened by two reviewers using the Rayyan web application for systematic reviews. First, articles will be screened by titles and/or abstracts. The screened articles will be classified into three groups: relevant, irrelevant, and uncertain. Articles that both reviewers consider to be irrelevant will be excluded from the review. For articles that are considered relevant or uncertain based on their title and/or abstract, the full text article will be retrieved to assess eligibility. Potentially relevant articles selected by at least one author will be investigated and evaluated in full. Next, the full text of the eligible articles will be reviewed and a list of articles to be included by each reviewer will be created. The two lists will then be compared, and the discrepancy will be discussed. If no agreement is reached, the entire team will make the final decision. A flow diagram showing the selection process will be created using the PRISMA guidelines.

For peer review only

For peer review only - http://bmjopen.bmj.com/site/about/guidelines.xhtml

Data extraction

Predefined information of interest from included studies will be extracted using a data extraction form. This will help standardize the information recorded and aid analyses. The following data will be extracted independently by two reviewers: study name (along with the name of the first author and year of publication), country where the study was conducted, study size, source from which patients or study participants were selected, study design, definition of AI, definition of CAH, disease duration, outcomes definition, age, gender, ethnicity, incidence or prevalence with 95% confidence intervals (CIs) or the number of patients achieving the outcomes. Data on causes of PAI and SAI will be systematically gathered, as underlying conditions themselves may independently influence the outcomes. Data on doses of CG and MC will be collected, as treatment strategies have changed over time. Studies including patients with hypopituitarism will be included with the aim to gather data on patients with SAI. If the required data are inadequate, unclear, or missing from the article, authors will be contacted. If needed, missing information will be calculated from the available data when possible. In case of studies with overlapping population, only the study with the largest sample size and higher quality will be included. The data extraction will be discussed by two reviewers, and the entire team will arbitrate in the case of any disagreement.

Quality assessment

The quality of the studies will be independently assessed by two authors using the Newcastle–Ottawa quality assessment scale (NOS). Critical appraisal of articles will be performed to assess the methodological quality of each article. The quality appraisal will be undertaken by two reviewers and a third reviewer will arbitrate in the case of any disagreement.

Data synthesis

Characteristics and main results of the included studies will be presented in descriptive tables and the gathered evidence will be synthesized narratively.

For peer review only

We will calculate rate of mortality in each study if possible. If the included studies are sufficiently homogeneous, we will undertake a meta-analysis by pooling estimates across studies using the random effects approach.

Heterogeneity between studies will be assessed using the Higgins I^2 statistic (18). I^2 values of 0–30% will be considered as minimal heterogeneity, 31–50% moderate heterogeneity, and > 50% substantial heterogeneity (18). As PAI and SAI are two different entities, we will conduct separate subanalyses on mortality in PAI and SAI. If data are sufficient, we will conduct subgroup analysis to explore potential reasons for heterogeneity based on the quality of study, country, age, gender, and ethnicity. A subgroup analysis will be also conduct for patients with CAH.

Begg's funnel plot and Egger's test will be used to assess publication bias (19). The former is a scatter plot of the log odds ratios (ORs) of individual studies on the x-axis against 1/ standard error (SE) of each study on the y-axis(19). Egger's test is a linear regression test of the normalized effect estimate (log OR/SE) against its precision (1/SE) (19). P value of < 0.10 on Egger's test or an asymmetrical funnel plot will be considered to indicate the presence of publication bias.

Ethics and dissemination

This systematic review does not require ethical approval or informed consent because it will be based on previously published data only and does not implicate any direct contact with individual patients. The research results will be presented at scientific conferences and submitted for publication in an internationally recognized peer-reviewed scientific journal.

Patient and public involvement

No patient will be involved in this systematic review

Discussion

For peer review only

Adrenal insufficiency is a life-threatening disorder. In the past, most patients with AI died within a few years after diagnosis due to scarce treatment options (3). Thanks to the discovery of cortisone in 1940s, the survival of patients with AI has improved (3). In the 1960s, Mason et al. showed that PAI patients on GC replacement therapy had a mortality rate similar to that of the background population (20). In line with that study, a recent Norwegian study, including 811 patients with PAI, showed no difference in mortality rate between PAI patients and the general Norwegian population (11). Conversely, our group showed that mortality in PAI patients was 2-fold increased in comparison with the general Swedish population, after following 1675 patients with PAI between 1987 and 2001 (6). More recent data from UK have confirmed this finding showing a more than 2-fold excess mortality in patients with PAI (16).
Mortality in AI patients has been related to cardiovascular diseases, infections, acute adrenal crises, and malignancies (7). In a large retrospective cohort study including 6821 patients with AI from United Kingdom, Ngaosuwan et al. have recently shown that overall mortality is higher by approximately 80% in PAI and 50% in SAI in comparison with matched controls (7). Another study on the same cohort has showed that mortality due to cardiovascular disease was increased in both PAI and SAI compared with matched controls (21). Interestingly, patients with SAI had an increased risk of cerebrovascular events when compared with PAI patients (21).

Other common causes of death in AI are infectious diseases (7). A multicenter study including 2034 patients with PAI and SAI from the European Adrenal Insufficiency Registry (EU-AIR) showed that the major causes of death were cardiovascular disease (35%) and infectious diseases (15%)(22). In a population-based study, including 3299 patients with PAI, Bensing *et al.* have found that the risk of death due to infections was 6-fold increased compared to the general population (5). Moreover, in a cohort study of 1286 hypopituitary patients, Burman *et al.* have found that mortality from infections was increased in ACTH-deficient patients but not in patients without ACTH deficiency (12).

Inappropriate replacement therapy with GCs has been described as the main determinant of a poor prognosis in AI patients (23). Excessive cortisol exposure and glucocorticoid replacement therapy that does not mimic cortisol circadian rhythm are indeed associated with the adverse metabolic profile, increased morbidity, and mortality (23, 24).

Over the last two decades, efforts have been made to optimize GC treatment, trying to reduce the total daily GC dose and optimise intercurrent illness management (9). In addition, novel

For peer review only - http://bmjopen.bmj.com/site/about/guidelines.xhtml

treatment strategies have been introduced to better mimic the physiological diurnal variation in cortisol levels (10). Whether this has led to an improvement in long-term outcomes remains unknown. Previous studies conducted on this topic have shown controversial findings due to different study designs, populations and settings (5, 6, 11). Since AI is a rare disease, no single center has accumulated sufficient experience to draw definitive conclusions. Therefore, a systematic review is needed to provide clearer insights into this topic. Increasing knowledge of AI mortality can lead to optimized management of AI and its comorbidities.

Contributions

DE conceived this study. FA, KD, DP and DE developed the study protocol. All the authors revised the study protocol. FA and KD will implement the systematic review under the supervision of DE, EB, RB, DC, BN, DP and GJ. EH will develop the search strings and conduct the study search. EB and BN will provide the statistical analysis expertise. EB, BN, and DE will conduct data analysis. FA and KD will perform the screening and extraction of data whereas DE, EB, RB, DC, BN and GJ will review the work.

Funding statement

This work received support from the Swedish state under the agreement between the Swedish government and the county councils, the ALF-agreement (ALFGBG-874631 and ALFGBG-960884).

Conflicts of interest and financial disclosure

None declared

For peer review only

References

1. Charmandari E, Nicolaides NC, Chrousos GP. Adrenal insufficiency. Lancet. 2014;383(9935):2152-67.
2. Husebye ES, Pearce SH, Krone NP, Kampe O. Adrenal insufficiency. Lancet. 2021;397(10274):613-29.
3. Kumar R, Wassif WS. Adrenal insufficiency. J Clin Pathol. 2022;75(7):435-42.
4. Dunlop D. Eighty-Six Cases of Addison's Disease. Br Med J. 1963;2(5362):887-91.
5. Bensing S, Brandt L, Tabaroj F, Sjoberg O, Nilsson B, Ekbom A, et al. Increased death risk and altered cancer incidence pattern in patients with isolated or combined autoimmune primary adrenocortical insufficiency. Clin Endocrinol (Oxf). 2008;69(5):697-704.
6. Bergthorsdottir R, Leonsson-Zachrisson M, Oden A, Johannsson G. Premature mortality in patients with Addison's disease: a population-based study. J Clin Endocrinol Metab. 2006;91(12):4849-53.
7. Ngaosuwan K, Johnston DG, Godsland IF, Cox J, Majeed A, Quint JK, et al. Increased Mortality Risk in Patients With Primary and Secondary Adrenal Insufficiency. J Clin Endocrinol Metab. 2021;106(7):e2759-e68.
8. Ngaosuwan K, Johnston DG, Godsland IF, Cox J, Majeed A, Quint JK, et al. Mortality Risk in Patients With Adrenal Insufficiency Using Prednisolone or Hydrocortisone: A Retrospective Cohort Study. J Clin Endocrinol Metab. 2021;106(8):2242-51.
9. Johannsson G, Skrtic S, Lennernas H, Quinkler M, Stewart PM. Improving outcomes in patients with adrenal insufficiency: a review of current and future treatments. Curr Med Res Opin. 2014;30(9):1833-47.
10. Johannsson G, Nilsson AG, Bergthorsdottir R, Burman P, Dahlqvist P, Ekman B, et al. Improved cortisol exposure-time profile and outcome in patients with adrenal insufficiency: a prospective randomized trial of a novel hydrocortisone dual-release formulation. J Clin Endocrinol Metab. 2012;97(2):473-81.
11. Erichsen MM, Lovas K, Fougner KJ, Svartberg J, Hauge ER, Bollerslev J, et al. Normal overall mortality rate in Addison's disease, but young patients are at risk of premature death. Eur J Endocrinol. 2009;160(2):233-7.
12. Burman P, Mattsson AF, Johannsson G, Hoybye C, Holmer H, Dahlqvist P, et al. Deaths among adult patients with hypopituitarism: hypocortisolism during acute stress, and de novo malignant brain tumors contribute to an increased mortality. J Clin Endocrinol Metab. 2013;98(4):1466-75.
13. O'Reilly MW, Reulen RC, Gupta S, Thompson CA, Dineen R, Goulden EL, et al. ACTH and gonadotropin deficiencies predict mortality in patients treated for nonfunctioning pituitary adenoma: long-term follow-up of 519 patients in two large European centres. Clin Endocrinol (Oxf). 2016;85(5):748-56.
14. Hammarstrand C, Ragnarsson O, Hallen T, Andersson E, Skoglund T, Nilsson AG, et al. Higher glucocorticoid replacement doses are associated with increased mortality in patients with pituitary adenoma. Eur J Endocrinol. 2017;177(3):251-6.
15. Gola M, Bonadonna S, Doga M, Giustina A. Clinical review: Growth hormone and cardiovascular risk factors. J Clin Endocrinol Metab. 2005;90(3):1864-70.
16. Conrad N, Verbeke G, Molenberghs G, Goetschalckx L, Callender T, Cambridge G, et al. Autoimmune diseases and cardiovascular risk: a population-based study on 19 autoimmune diseases and 12 cardiovascular diseases in 22 million individuals in the UK. Lancet. 2022;400(10354):733-43.

For peer review only - http://bmjopen.bmj.com/site/about/guidelines.xhtml

17. Shamseer L, Moher D, Clarke M, Ghersi D, Liberati A, Petticrew M, et al. Preferred reporting items for systematic review and meta-analysis protocols (PRISMA-P) 2015: elaboration and explanation. BMJ. 2015;350:g7647.
18. Higgins JP, Thompson SG. Quantifying heterogeneity in a meta-analysis. Stat Med. 2002;21(11):1539-58.
19. Egger M, Smith GD. Bias in location and selection of studies. BMJ. 1998;316(7124):61-6.
20. Mason AS, Meade TW, Lee JA, Morris JN. Epidemiological and clinical picture of Addison's disease. Lancet. 1968;2(7571):744-7.
21. Ngaosuwan K, Johnston DG, Godsland IF, Cox J, Majeed A, Quint JK, et al. Cardiovascular Disease in Patients With Primary and Secondary Adrenal Insufficiency and the Role of Comorbidities. J Clin Endocrinol Metab. 2021;106(5):1284-93.
22. Quinkler M, Ekman B, Zhang P, Isidori AM, Murray RD, Investigators E-A. Mortality data from the European Adrenal Insufficiency Registry-Patient characterization and associations. Clin Endocrinol (Oxf). 2018;89(1):30-5.
23. Rahvar AH, Haas CS, Danneberg S, Harbeck B. Increased Cardiovascular Risk in Patients with Adrenal Insufficiency: A Short Review. Biomed Res Int. 2017;2017:3691913.
24. Esposito D, Bobbio E, Di Fraia R, Mone P, Accardo G, De Bellis A, et al. Patients with adrenal insufficiency have cardiovascular features associated with hypovolemia. Endocrine. 2020;70(2):412-20.

For peer review only

For peer review only - http://bmjopen.bmj.com/site/about/guidelines.xhtml

1

PRISMA-P 2015 Checklist

This checklist has been adapted for use with protocol submissions to *Systematic Reviews* from Table 3 in Moher D et al: Preferred reporting items for systematic review and meta-analysis protocols (PRISMA-P) 2015 statement. *Systematic Reviews* 2015 **4**:1

Section/topic	#	Checklist item	Information reported		Line number(s)
			Yes	No	
**ADMINISTRATIVE INFORMATION**					
**Title**					
Identification	1a	Identify the report as a protocol of a systematic review	X		
Update	1b	If the protocol is for an update of a previous systematic review, identify as such		X	
**Registration**	2	If registered, provide the name of the registry (e.g., PROSPERO) and registration number in the Abstract	X		
**Authors**					
Contact	3a	Provide name, institutional affiliation, and e-mail address of all protocol authors; provide physical mailing address of corresponding author	X		
Contributions	3b	Describe contributions of protocol authors and identify the guarantor of the review	X		
**Amendments**	4	If the protocol represents an amendment of a previously completed or published protocol, identify as such and list changes; otherwise, state plan for documenting important protocol amendments		X	
**Support**					
Sources	5a	Indicate sources of financial or other support for the review	X		
Sponsor	5b	Provide name for the review funder and/or sponsor	X		
Role of sponsor/funder	5c	Describe roles of funder(s), sponsor(s), and/or institution(s), if any, in developing the protocol		X	
**INTRODUCTION**					
**Rationale**	6	Describe the rationale for the review in the context of what is already known	X		
**Objectives**	7	Provide an explicit statement of the question(s) the review will address with reference to participants, interventions, comparators, and outcomes (PICO)	X		
**METHODS**					

BioMed Central The Open Access Publisher

Section/topic	#	Checklist item	Information reported Yes	No	Line number(s)
**Eligibility criteria**	8	Specify the study characteristics (e.g., PICO, study design, setting, time frame) and report characteristics (e.g., years considered, language, publication status) to be used as criteria for eligibility for the review	X		
**Information sources**	9	Describe all intended information sources (e.g., electronic databases, contact with study authors, trial registers, or other grey literature sources) with planned dates of coverage	X		
**Search strategy**	10	Present draft of search strategy to be used for at least one electronic database, including planned limits, such that it could be repeated		X	
STUDY RECORDS					
Data management	11a	Describe the mechanism(s) that will be used to manage records and data throughout the review	X		
Selection process	11b	State the process that will be used for selecting studies (e.g., two independent reviewers) through each phase of the review (i.e., screening, eligibility, and inclusion in meta-analysis)	X		
Data collection process	11c	Describe planned method of extracting data from reports (e.g., piloting forms, done independently, in duplicate), any processes for obtaining and confirming data from investigators	X		
**Data items**	12	List and define all variables for which data will be sought (e.g., PICO items, funding sources), any pre-planned data assumptions and simplifications	X		
**Outcomes and prioritization**	13	List and define all outcomes for which data will be sought, including prioritization of main and additional outcomes, with rationale	X		
**Risk of bias in individual studies**	14	Describe anticipated methods for assessing risk of bias of individual studies, including whether this will be done at the outcome or study level, or both; state how this information will be used in data synthesis	X		
DATA					
**Synthesis**	15a	Describe criteria under which study data will be quantitatively synthesized	X		
	15b	If data are appropriate for quantitative synthesis, describe planned summary measures, methods of handling data, and methods of combining data from studies, including any planned exploration of consistency (e.g., I^2, Kendall's tau)	X		
	15c	Describe any proposed additional analyses (e.g., sensitivity or subgroup analyses, meta-regression)	X		
	15d	If quantitative synthesis is not appropriate, describe the type of summary planned		X	
**Meta-bias(es)**	16	Specify any planned assessment of meta-bias(es) (e.g., publication bias across studies, selective reporting within studies)	X		
**Confidence in cumulative evidence**	17	Describe how the strength of the body of evidence will be assessed (e.g., GRADE)	X		

BioMed Central The Open Access Publisher
